# Molecular Phylogeny of *Uropsilus* (Talpidae, Eulipotyphla, Mammalia) With a New Species Described From Henan Province, China

**DOI:** 10.1002/ece3.70928

**Published:** 2025-02-12

**Authors:** Haijun Jiang, Yaohua Yang, Yanyan Zhou, Jundong Tian, Jiqi Lu

**Affiliations:** ^1^ School of Life Sciences Zhengzhou University Zhengzhou China; ^2^ Institute of Biodiversity and Ecology Zhengzhou University Zhengzhou China

**Keywords:** China, Eulipotyphla, Henan Province, morphology, phylogeny, *Uropsilus funiushanensis* sp. nov

## Abstract

Shrew moles (*Uropsilus*), belonging to subfamily Uropsilinae of family Talpidae, were predominantly found in Myanmar, Vietnam, Bhutan, and Southwestern China. However, the extent distribution range of species within *Uropsilus* remains unclear. In 2022, we collected 18 specimens of *Uropsilus* from the western mountains of Henan Province in central China. Based on the mitochondrial (CYT B) and nuclear (RAG1 + RAG2) genes, the phylogenetic relationships, divergence times, and species delimitation of *Uropsilus* were analyzed. The results showed that the genetic distance of CYT B between new species and other species of *Uropsilus* ranged from 8.44% to 18.23%; the new species was a distinct valid species and speciated in the early Pleistocene (2.16 Ma, 95% CI = 2.00–2.65) based on phylogenetic analyses. Morphologically, the new species was distinguishable from the other species of *Uropsilus* by its yellowish–brown dorsum and ash‐black venter, short tail, slender and curved zygomatic arch, upper inward‐curved I1, and lack of a gap between I1 and I2. The combined results of morphological and molecular phylogenetic analysis indicated that the samples from Henan Province represented a new species—*Uropsilus funiushanensis* sp. nov. The specimens collected from the mountainous area of western Henan Province and Shennongjia in Hubei Province were identified as the same species. We suggested the evolutionary mechanism of the dental formula of *U. funiushanensis* and *U. soricilus* groups should be explored in the future.

## Graphical abstract

A new species is revealed based on molecular phylogenetic and morphological analysis. The result from this study indicated that the species diversity of Eulipotyphla in Henan province must have been underestimated previously.

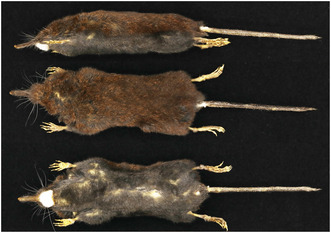

## 1 Introduction

Shrew moles (*Uropsilus* spp.), belonging to subfamily Uropsilinae, family Talpidae, order Eulipotyphla, distribute mainly in grassland and forest environments from altitudes 1064 to 4600 m in the mountainous areas of Myanmar, Northern Vietnam, and southwestern China, as well as the Mts. Dabieshan in central China and Wuyishan in eastern China (Bui et al. 2023; Hoffmann 1984; Hu et al. 2021; Kryštufek and Motokawa 2018; Ren et al. 2023; Wan 2015; Wan, He, and Jiang 2013; Wan et al. 2018; Wei et al. 2021). The extant species of Uropsilinae consist solely of the genus *Uropsilus* (Smith and Xie 2008; Wan 2015; Wan, He, and Jiang 2013; Wan et al. 2018; Wei et al. 2021). To date, no species of *Uropsilus* have been reported from Henan Province, China. Shrew moles have retained the terrestrial habits and their morphological characteristics of primitive moles, including slender front claws, exposed external ears, and tails close to body length (Allen 1938). Morphological and molecular phylogenetic analysis consistently identify Uropsilinae as the most ancient group of the Talpidae family (Douady and Douzery 2003; He et al. 2017; Motokawa 2004; Sanchez‐Villagra, Horovitz, and Motokawa 2006; Shinohara, Campbell, and Suzuki 2003).

The classification of Uropsilinae has undergone several major adjustments in previous studies based on varied dental formulae. The dental formulas of extant *Uropsilus* species are shown in Table S1. The first recognized species of Uropsilinae was *U. soricipes* , which was described based on the specimens from Baoxing (= Muping) in Sichuan Province (Milne‐Edwards 1871). Subsequently, Thomas recognized two genera (*Rhynchonax* and *Nasillus*) and three species (*Rh. andersoni* 1912, *N. gracilis* 1912, and *N. investigator* 1922) of Uropsilinae from China based on differences in dental formulas (Thomas 1912, 1922). Additionally, *Rh. a. atronates* and *Rh. a. nivatus* were described based on the specimens from the Salween drainage and Mt. Yulongxueshan area in Yunnan Province, southwest China (Allen 1923). The subfamily Uropsilinae includes three genera, four species, and six subspecies (Allen 1938). Ellerman and Morrison‐Scott (1951) classified three genera (*Uropsilus*, *Rhynchonax*, and *Nasillus*) under *Uropsilus* into one species ( *U. soricipes* ) and five subspecies (*U*. *s*. *soricipes*, *U. s. gracilis*, *U*. *s*. *andersoni*, *U. s. investigator*, and *U. s. nivatus*). Hoffmann (1984) recognized three species ( *U. soricipes* , *U. gracilis* , and *U. andersoni* ) based on their different dental formulas. Wang and Yang (1989) argued that *U. investigator* should be classified as an independent species due to its sympatric distribution with *U. gracilis* . Since then, the subfamily Uropsilinae has been widely accepted to include one genus with four species (Hutterer 2005; Smith and Xie 2008).

Recently, several new species (including putative species) of *Uropsilus* have been identified and described. Liu et al. (2013) described *U. aequodonenia* from Mt. Luojishan, Sichuan Province. Wan, He, and Jiang (2013) identified seven recognized species as well as three putative species based on molecular approaches. Latterly, Wan et al. (2018) described seven recognized species and eight putative species based on molecular analyses. Additionally, Hu et al. (2021) described a new species, *U. dabieshanensis*, based on molecular and morphological data analysis from Mt. Dabieshan, Anhui Province, central China. More recently, two new species were described based on morphological and molecular data analysis in 2023: *U*. *fansipanensis* from northwestern Vietnam (Bui et al. 2023) and *U*. *huanggangensis* from Mt. Huanggangshan, Jiangxi Province, eastern China (Ren et al. 2023). To date, the genus‐level classification of shrew moles has not been revised, with 10 recognized species (*U. aequodonenia*, *U. andersoni* , *U. atronates*, *U. dabieshanensis*, *U. fansipanensis*, *U. gracilis* , *U. huanggangensis*, *U. investigator* , *U. nivatus*, and *U. soricipes* ) and eight putative species reported within *Uropsilus* (Bui et al. 2023; Hu et al. 2021; Ren et al. 2023; Wan 2015; Wan, He, and Jiang 2013; Wan et al. 2018).

Shrew moles originated in the southern Mt. Hengduanshan area and further expanded their distribution. Speciation in *Uropsilus* occured primarily through allopatric speciation due to limited dispersal ability. The complex topography of the southwestern mountains leads to the geographical isolation of *Uropsilus* species and promotes genetic differentiation among those species. Moreover, ancestral habitat preferences and the suitable environments provided by the southwestern mountains have contributed to high morphological similarity, contributing to the emergence of cryptic diversity within *Uropsilus* (Wan 2015; Wan et al. 2018). With the discovery of new *Uropsilus* species in central and eastern China, it is obvious that the distribution ranges have been underestimated, much less the limited knowledge about the dispersal route of the ancestral groups between southwestern and central‐eastern China.

From April to September 2022, a total of 18 specimens of *Uropsilus* were collected from the western mountainous areas of Henan Province, central China. Based on morphological features and measurements, and combined with molecular analysis, we intended to identify the specific status of these specimens and aimed to contribute basic information to the mammalian species dataset of Henan Province and China.

## 2 Materials and Methods

### 2.1 Sampling and Sequencing

**FIGURE 1:**
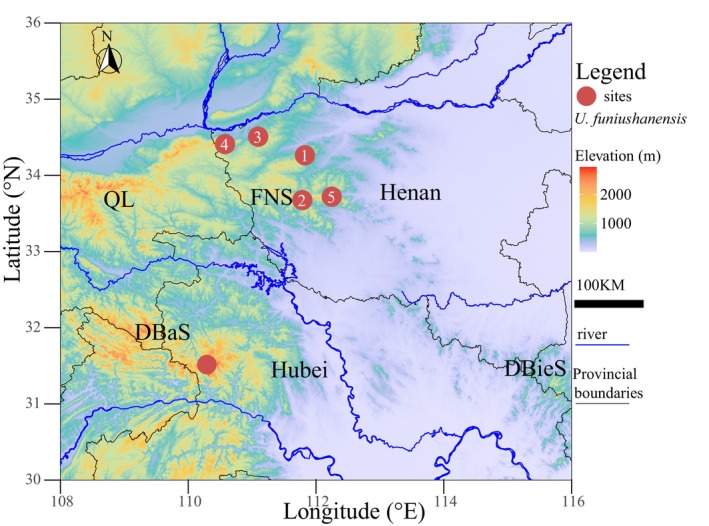
Sampling localities of *U. funiushanensis* sp. nov. in Henan and Hubei Provinces. DBaS, Mt. Dabashan; DBieS, Mt. Dabieshan; FNS, Mt. Funiushan; QL, Mt. Qinling.

In 2022, 18 specimens of *Uropsilus* were collected from Xiaoqinling of Lingbao, Yanzishan of Lingbao, Yaoshan of Lushan, Tianchishan of Songxian, and Longyuwan of Luanchuan in Henan Province, central China. The pitfall traps (plastic buckets, 15 cm in diameter and 28 cm in depth) and rat clips (12 cm in length and 7 cm in width) were placed in various habitats (including crevices in deciduous broad‐leaved forests, animal pathways, and the humus layer) for sampling (Figure 1 and Table S2). Voucher specimens were deposited at the Institute of Biodiversity and Ecology (IBE), Zhengzhou University. Muscle and liver tissue was collected and preserved in 95% ethanol and stored at −80°C.

Total genomic DNA of the 18 specimens was extracted from the liver and muscle tissues using the DNA extraction kit (TIANamp Genomic DNA Kit, Beijing, China). The primers of the mitochondrial gene (Cytochrome B, CYT B) and three nuclear gene segments, including Phospholipase C Beta 4 (PLCB4), Recombination activating gene 1 (RAG1), and Recombination activating gene 2 (RAG2), were sequenced, following Wan (2015) (Table S3). The 25 μL PCR amplification system, consisting of mix (22 μL), forward primer (1 μL), reverse primer (1 μL), and DNA (1 μL). The mix used was GoldenStar T6 Super PCR Mix Ver.2. PCR products were sequenced at Sangon Biotech Co. Ltd. Sequencing results were compared using MEGA 11 (Tamura, Stecher, and Kumar 2021).

### 2.2 Phylogenetic Analyses

A total of 64 specimens were analyzed, including 18 specimens sequenced and 46 specimens of *Uropsilus* downloaded from NCBI (Table S4). The best‐fit evolutionary models were selected by the Akaike Information Criterion (AIC) using jModeltest v2.1.10 (Table S5) (Darriba et al. 2012). The phylogeny was reconstructed using Bayesian inference (BI) based on five different datasets (dataset 1: CYT B, dataset 2: RAG1 + RAG2, dataset 3: RAG1, dataset 4: RAG2, dataset 5: PLCB4). The BI tree was constructed using BEAST 1.10.4 (Suchard et al. 2018) and employed relaxed uncorrelated exponential clock models, birth‐death process tree priors, and the default prior distribution of the program for the model parameters. Each analysis was run for 50 million generations and sampled every 5000 generations. Convergence was assessed using Tracer v1.7 to confirm that the effective sample sizes (ESS) were greater than 200 (Rambaut et al. 2018). RAG1 and RAG2 were selected to construct the nuDNA dataset, as the phylogenetic analysis of the PLCB4 did not clarify the phylogenetic relationships of *Uropsilus*.

### 2.3 Molecular Dating

According to the BI results, the dataset (CYT B + RAG1 + RAG2) was selected for divergence time estimated using the birth‐death model and relaxed lognormal model in BEAST v1.10.4 (Suchard et al. 2018). Each analysis was run for 100 million generations and sampled every 5000 generations. The fossil calibration age constraints for the time settings followed Wan et al. (2018). Specifically, (1) a normal distribution (mean = 6.18 Ma, standard deviation = 1.5 Ma) was used as the prior for estimating the divergence time of *Uropsilus*, based on the first appearance of its ancestors in the late Miocene; (2) the earliest known *U. soricipes* from the Early Pleistocene (2.0–2.4 Ma) was used to set an exponential prior with a boundary at 2.0 Ma (offset = 2.0, mean = 0.67).

### 2.4 Species Delimitation

Three methods for species delimitation were selected as follows. (1) The Kimura 2‐Parameter (K2P) genetic distances between species of *Uropsilus* were calculated using MEGA 11 based on CYT B (Tamura, Stecher, and Kumar 2021). (2) Assemble Species by Automatic Partitioning (ASAP) was used for species delimitation analyses based on CYT B (Puillandre, Brouillet, and Achaz 2021). The Maximum Likelihood Estimate of Transition (TS)/Transversion (TV) bias for *Uropsilus* was calculated using MEGA 11 (Kimura 1980; Tamura, Stecher, and Kumar 2021). The Kimura (K80) TS/TV was selected based on CYT B for the ASAP analysis using the webserver (https://bioinfo.mnhn.fr/abi/public/asap/asapweb.html). (3) The Bayesian Phylogenetics and Phylogeography (BPP) analyses were used to define the species within the genus *Uropsilus* (Yang and Rannala 2014). The parameter settings for BPP followed Jiang et al. (2023). The validity of our assignment of *Uropsilus* species was evaluated via BPP v3.1 with nuDNA (RAG1 + RAG2) data from 64 specimens (18 species), which were included in the species delimitation analysis. Each rjMCMC was run for 100,000 generations, sampling every 100 generations, and the 10,000 generations were discarded as burn‐in.

### 2.5 Morphological Measurements and Analyses

In total, 31 specimens of *Uropsilus* were examined and assigned to the following species: *U*. *dabieshanensis* (*n* = 4), *U*. *funiushanensis* sp. nov. (*n* = 12), *U. gracilis* (*n* = 5), *U. soricipes* (*n* = 7), and *U*. sp. 5 (*n* = 3). External morphology data, including head and body length (hbl), tail length (tl), and hindfoot length (hl), were measured with a ruler to the nearest 0.1 mm. Ten craniodental measurements were taken using digital calipers, including profile length (PL), basal length (BL), zygomatic breadth (ZB), least breadth between orbits (LBO), height of braincase (HB), median palatal length (MPL), greatest width at the anterior labial margins of the second mandibular molars (M^2^–M^2^), mandibular length (ML), lower tooth row length (LTRL), and upper tooth row length (UTRL) (Yang et al. 2007, 2005). The external morphology and dental formula of the new species were compared with other species within *Uropsilus* (including *U*. sp. 5, *U. dabieshanensis*, *U. gracilis* , *U. soricipes* , *U. investigator* , *U. andersoni* , *U. aequodonenia*, *U. atronates*, *U. nivatus*, *U. fansipanensis*, and *U. huanggangensis*).

The statistical analysis of cranial measurement data was conducted using R version 4.4.1 (R Core Team 2022) and RStudio version 2024.04.2 (R Studio Team 2024). Four species ( *U. gracilis* , *U. soricipes* , *U. dabieshanensis* and *U*. sp. 5) were selected for Principal Component Analysis (PCA) and Linear Discriminant Analysis (LDA). Shrew moles primarily undergo speciation through allopatric processes due to significant geographic isolation, and these species are geographically closest to the *U*. *funiushanensis* sp. nov. The BI results (CYT B, RAG1, and nuDNA) also revealed a closer genetic relationship between *U*. *funiushanensis* sp. nov. and three species (except for *U. gracilis* ). (1) Before conducting the PCA and LDA, analysis of variance (ANOVA) was performed on the cranial measurement data of the 31 specimens using the ‘anova’ function in R version 4.4.1. (2) Discriminant analysis and principal component analysis were further conducted on the samples based on the craniodental variables. The overall similarities of skull morphology were assessed using PCA. PCA mainly includes the following steps: (i) read the data and convert it into a data frame; (ii) standardize the data by centering and scaling the selected columns; (iii) perform PCA analysis based on the ‘prcomp’ function; (iv) extract and average PCA results; (v) calculate centroids and prepare data for plotting; (vi) plot PCA results using ‘ggplot2’. (3) LDA was applied to differentiate between groups of individuals with similar morphologies, and steps as follows: (i) data standardization; (ii) LDA was performed using the ‘lda’ function from the ‘MASS’ package; (iii) the results from LDA were extracted and saved; (iv) the results of LDA were visualized using the ‘ggplot2’ package. All statistical tests were useing the two‐tailed, and *p* < 0.05 for the level of significance.

## 3 Results

### 3.1 Phylogenetic Analyses, Divergence Time, and Species Delimitation

We obtained 3070 bp sequences for each voucher specimen, including 1140 bp mitochondrial [CYT B (1140 bp), GenBank ID: PP430752–PP430769] and 1930 bp nuclear [PLCB4 (276 bp), GenBank ID: PP430770–PP430788; RAG1 (995 bp), GenBank ID: PP430789–PP430806; RAG2 (659 bp), GenBank ID: PP430807–PP430824] sequences.

**FIGURE 2:**
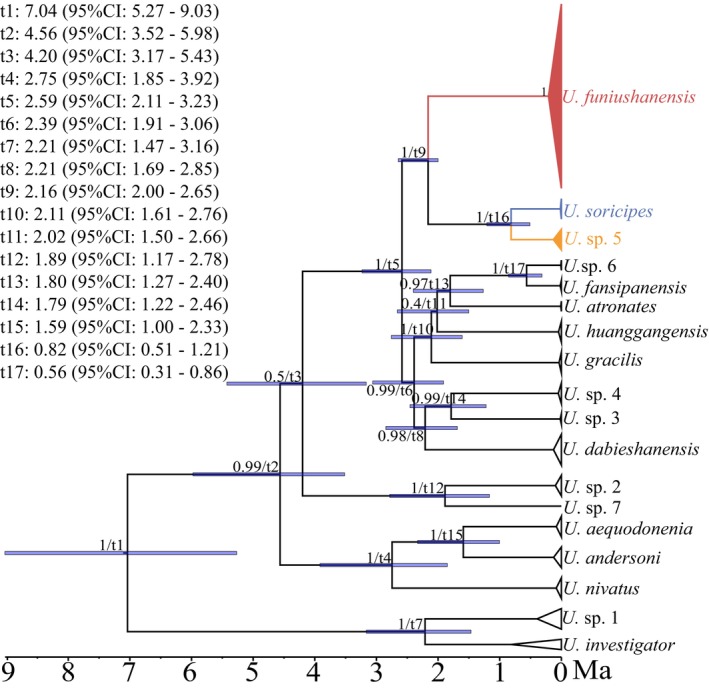
Divergence times of *Uropsilus* estimated based on CYT B + RAG1 + RAG2. Numbers at each node represent posterior probabilities/divergence times.

**FIGURE 3:**
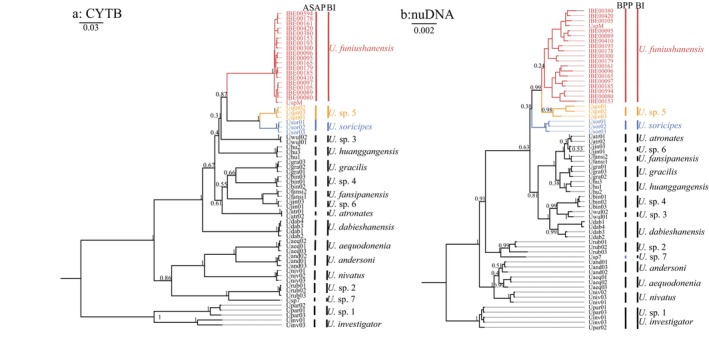
Bayesian inference analyses results of *Uropsilus* based on CYT B (a) and nuDNA (RAG1 + RAG2, b). Branch numbers refer to BI posterior probabilities (PP).

The result derived from divergence time estimation based on CYTB + RAG1 + RAG2 was shown in Figure 2. The results of divergence time estimates showed that *U. funiushanensis* sp. nov. and *U. soricipes* group (*U*. sp. 5 + *U. soricipes* ) diverged at 2.16 Ma (95% CI = 2.00–2.65), with PP = 1.00 (Figure 2). The Most BI results [CYT B (PP = 1.00), nuDNA (RAG1 + RAG2, PP = 0.24), and RAG2 (PP = 0.3)] indicated *U. funiushanensis* sp. nov. as a distinct species (Figure 3, S1a). In contrast, the RAG2 results showed that *U. funiushanensis* sp. nov. was clustered with *U*. sp. 5 and *U*. soricipes (Figure S1b). The phylogenetic result for PLCB4 did not clearly resolve the phylogenetic relationships of *Uropsilus*, although it separates *U. funiushanensis* sp. nov. from *U. soricipes* and *U*. sp. 5 (Figure S1c).

Compared to the other species within *Uropsilus*, the K2P distances of *U*. *funiushanensis* sp. nov. ranged from 8.44% (*U*. sp. 5) to 18.23% ( *U. investigator* ) (Table 1). The ASAP analyses generated 10 partitions, with the optimal partition identifying 16 species within the genus *Uropsilus* (Figure S2). In this optimal partition, *U*. *funiushanensis* sp. nov. was identified as a distinct species, while *U. soricipes* and *U*. sp. 5 were classified as a single species. The BPP analysis revealed the existence of multiple species within the genus *Uropsilus*, with estimates ranging from 9 to 18 species across 12 different models. Notably, 75% of the results supported delimitation of 16 species results within the genus *Uropsilus* (Figure 3b, Table 2). All optimal results supported *U. funiushanensis* sp. nov. as a distinct species, with PP > 0.95 (Table 2).

**TABLE 1:** K2P distances between species of *Uropsilus* based on CYT B.

	*U. funiu*	*U. inv*	*U. gra*	*U. sor*	*U. aeq*	*U. and*	*U. niv*	*U. atr*	*U*. sp. 6	*U*. sp. 1	*U*. sp. 4	*U*. sp. 2	*U*. sp. 5	*U*. sp. 3	*U*. sp. 7	*U. dab*	*U. hu*
*U. inv*	18.23%																
*U. gra*	9.61%	18.91%															
*U. sor*	8.75%	17.57%	9.39%														
*U. aeq*	15.09%	19.95%	14.82%	13.44%													
*U. and*	14.99%	19.45%	15.51%	14.80%	8.43%												
*U. niv*	13.79%	18.32%	12.91%	14.03%	11.30%	10.43%											
*U. atr*	11.07%	19.91%	10.86%	10.51%	16.35%	16.61%	15.11%										
*U*. sp. 6	10.06%	19.41%	9.67%	9.20%	16.37%	16.13%	14.68%	10.14%									
*U*. sp. 1	17.62%	10.57%	17.63%	17.46%	17.19%	18.29%	17.25%	17.98%	20.09%								
*U*. sp. 4	8.56%	18.60%	7.67%	9.17%	15.02%	14.59%	12.65%	10.53%	8.18%	18.15%							
*U*. sp. 2	14.39%	18.51%	14.70%	14.30%	15.00%	13.53%	14.48%	15.88%	13.54%	18.35%	14.08%						
*U*. sp. 5	8.44%	17.40%	9.88%	3.88%	14.43%	15.30%	14.46%	11.28%	9.17%	17.36%	9.38%	14.93%					
*U*. sp. 3	9.93%	17.69%	11.40%	10.68%	15.57%	15.49%	13.94%	11.88%	9.94%	17.77%	9.66%	14.68%	10.46%				
*U*. sp. 7	14.11%	19.80%	13.21%	14.94%	13.03%	13.85%	12.85%	14.64%	15.24%	18.38%	14.73%	8.80%	15.32%	14.94%			
*U. dab*	11.30%	18.26%	10.91%	11.67%	16.91%	16.50%	15.75%	14.78%	12.16%	17.90%	13.26%	14.06%	10.72%	13.07%	15.82%		
*U. hu*	10.19%	19.22%	10.30%	9.83%	15.61%	16.26%	15.18%	10.93%	9.49%	18.43%	9.47%	14.76%	10.63%	11.00%	15.51%	11.94%	
*U. fansi*	9.06%	20.72%	9.61%	9.18%	16.05%	15.88%	14.04%	9.52%	3.55%	19.39%	8.33%	13.87%	9.58%	11.05%	14.61%	11.99%	9.73%

**TABLE 2:** BPP results for *Uropsilus* based on nuDNA (RAG1 + RAG2).

q~G (2, 2000) and τ~G (2, 2000)						
nuDNA‐algorithm0	Species delimitation = 1 0 2	Species delimitation = 1 0 10	Species delimitation = 1 0 20	Species delimitation = 1 0 2	Species delimitation = 1 0 10	Species delimitation = 1 0 20
	Heredity = 1 1 4	Heredity = 1 1 4	Heredity = 1 1 4	Locusrate = 1 10.0	Locusrate = 1 10.0	Locusrate = 1 10.0
	PP (*U. funiushanensis*) = 1.00; P[12] = 0.00015; P[13] = 0.00085; P[14] = 0.05740; P[15] = 0.23265; **P[16] = 0.57610**; P[17] = 0.13165; P[18] = 0.00120	PP (*U. funiushanensis*) = 1.00; P[13] = 0.00295; P[14] = 0.05735; P[15] = 0.23425; **P[16] = 0.57745**; P[17] = 0.12165; P[18] = 0.00635	PP (*U. funiushanensis*) = 1.00; P[11] = 0.00125; P[12] = 0.00065; P[13] = 0.00405; P[14] = 0.06110; P[15] = 0.25400; **P[16] = 0.55865**; P[17] = 0.11450; P[18] = 0.00580	PP (*U. funiushanensis*) = 1.00; P[13] = 0.00105; P[14] = 0.03935; P[15] = 0.22060; **P[16] = 0.61985**; P[17] = 0.11585; P[18] = 0.00330	PP (*U. funiushanensis*) = 1.00; P[13] = 0.00165; P[14] = 0.04725; P[15] = 0.21540; **P[16] = 0.60530**; P[17] = 0.12505; P[18] = 0.00535	PP (*U. funiushanensis*) = 0.97; P[10] = 0.00665; P[11] = 0.03035; P[12] = 0.12060; P[13] = 0.28915; **P[14] = 0.33095**; P[15] = 0.16775; P[16] = 0.04795; P[17] = 0.00645; P[18] = 0.00015
nuDNA‐algorithm1	Species delimitation = 1 1 1 0.5	Species delimitation = 1 1 1.5 1	Species delimitation = 1 1 2 2	Species delimitation = 1 1 1 0.5	Species delimitation = 1 1 1.5 1	Species delimitation = 1 1 2 2
	Heredity = 1 1 4	Heredity = 1 1 4	Heredity = 1 1 4	Locusrate = 1 1 4	Locusrate = 1 1 4	Locusrate = 1 1 4
	PP (*U. funiushanensis*) = 1.00; P[12] = 0.00050; P[13] = 0.00570; P[14] = 0.12205; P[15] = 0.23705; **P[16] = 0.51380**; P[17] = 0.11460; P[18] = 0.00630	PP (*U. funiushanensis*) = 1.00; P[13] = 0.00120; P[14] = 0.04705; P[15] = 0.24120; **P[16] = 0.59030**; P[17] = 0.11350; P[18] = 0.00675	PP (*U. funiushanensis*) = 1.00; P[13] = 0.00195; P[14] = 0.06625; P[15] = 0.23580; **P[16] = 0.56180**; P[17] = 0.12885; P[18] = 0.00535	PP (*U. funiushanensis*) = 0.99; P[9] = 0.00045; P[10] = 0.00905; P[11] = 0.02730; P[12] = 0.15855; **P[13] = 0.34065**; P[14] = 0.28205; P[15] = 0.13860; P[16] = 0.03630; P[17] = 0.00705	PP (*U. funiushanensis*) = 1.00; P[10] = 0.00135; P[11] = 0.02040; P[12] = 0.10920; P[13] = 0.25550; **P[14] = 0.33250**; P[15] = 0.17885; P[16] = 0.08330; P[17] = 0.01825; P[18] = 0.00060	PP (*U. funiushanensis*) = 0.99; P[9] = 0.00075; P[10] = 0.00890; P[11] = 0.03880; P[12] = 0.11250; P[13] = 0.28775; **P[14] = 0.31135**; P[15] = 0.16310; P[16] = 0.06230; P[17] = 0.01280; P[18] = 0.00175

*Note:* Bold indicates the optimal results.

### 3.2 Morphological Statistical Analysis

**FIGURE 4:**
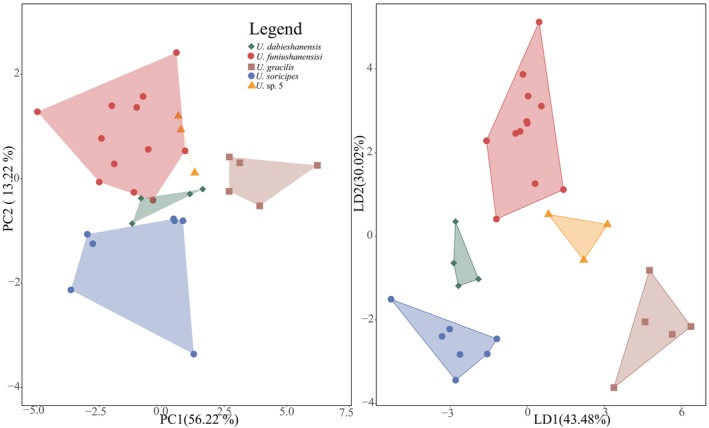
Results of principal component analysis (PCA) and linear discriminant analysis (LDA) for partial species of *Uropsilus* (*U*. *funiushanensis* sp. nov., *U. soricipes* , *U*. sp. 5, *U. gracilis*, and *U. dabieshanensis*).

Based on skull measurement indices, *U. funiushanensis* sp. nov. was distinguished from other species of *Uropsilus* (Table S6). Significant differences were observed among the eight measured indices: PL, LBO, HB, MPL, ZB, UTRL, ML, and LTRL (Table 3, Figure S3, *p* < 0.05) from the results of ANOVA. The PCA produced two axes (PC1 and PC2), which explained 56.22% and 13.22% of the variance (a total of 69.44%) (Table 3). The PC1 had the highest loadings for PL, BL, MPL, UTRL, ML, and LTRL, suggesting greater contributions from the length measurements (loading > 0.3). PC2 was highly positively correlated with HB, M^2^–M^2^ and ML (loading > 0.3), while ZB (−0.28) had a significant negative impact. The PCA plot showed that certain individuals of *U*. *funiushanensis* sp. nov. exhibit overlap with *U*. sp. 5 and *U. dabieshanensis*; however, most specimens can be distinguished from other species (Figure 4). The LDA results show that 31 specimens of *Uropsilus* were classified into five species (*U. soricpes*, *U. gracilis* , *U*. sp. 5, *U. dabieshanensis*, and *U. funiushanensis* sp. nov., respectively) (Figure 4).

**TABLE 3:** Character loadings and variance explained for PCA and LDA of *Uropsilus* ( *U. soricipes* , *U. gracilis* , *U*. sp. 0.5, *U. dabieshanensis* and *U. funiushanensis* sp. nov.).

Variables	PC1	PC2	LD1	LD2
PL	0.37	0.03	1.07	−0.85
BL	0.36	−0.20	−1.66	0.10
LBO	0.13	−0.70	−1.66	−1.23
HB	0.18	0.43	0.17	0.47
MPL	0.38	−0.15	0.73	−0.52
ZB	0.29	−0.28	1.28	0.01
UTRL	0.39	0.00	−0.25	−0.16
M^2^–M^2^	0.23	0.30	−0.80	−0.15
ML	0.35	0.32	0.10	1.76
LTRL	0.37	0.00	2.01	−1.18
PL	0.37	0.03	1.07	−0.85
Variance explained (%)	56.22	13.22	43.48	37.50

Abbreviations: BL, basal length; HB, height of braincase; LBO, least breadth between the orbits; LTRL: lower tooth row length; M^2^–M^2^, greatest width measured at anterior labial margins of second mandibular; ML, mandibular length; MPL, median palatal length; PL, profile length; UTRL, upper tooth row length; ZB, zygomatic breadth.

According to morphological features, molecular phylogenetic analyses, and species delimitation, *U. funiushanensis* sp. nov. was clearly distinguishable from other species of *Uropsilus*. We have reasonable grounds to describe and recognize these specimens from the western mountains of Henan Province as a new species in *Uropsilus*.

## 4 Taxonomic Account

*Uropsilus funiushanensis* Jiang & Lu sp. nov. https://zoobank.org/NomenclaturalActs/C2F92DBC‐1A7B‐4E0B‐AA54‐CAAD3395A03C.

Suggested common name. Funiushan shrew mole; Chinese common name: 伏牛山鼩鼹.

### 4.1 Type Materials

Holotype: IBE00300, an adult male collected by Haijun Jiang and Yaohua Yang in July 2022 from Yanzishan of Lingbao, Henan Province, China (WGS84 geodetic coordinate system, 34.507647 N, 111.095639 E, altitude 889.18 m a.s.l.). The skull and specimens were stored at the Institute of Biodiversity and Ecology, Zhengzhou University.

Paratypes: IBE00089, IBE00420, IBE00594. In 2022, a total of 18 specimens were collected from western mountains in Henan Province, and all specimens have been deposited in the Institute of Biodiversity and Ecology, Zhengzhou University.

Etymology. The specific name “*funiushanensis*” is derived from the Mt. Funiushan, the site where specimens were collected in this study and the type locality of the new species; the Latin adjectival suffix‐*ensis*‐denotes “belonging to.”

### 4.2 Diagnosis

**FIGURE 5:**
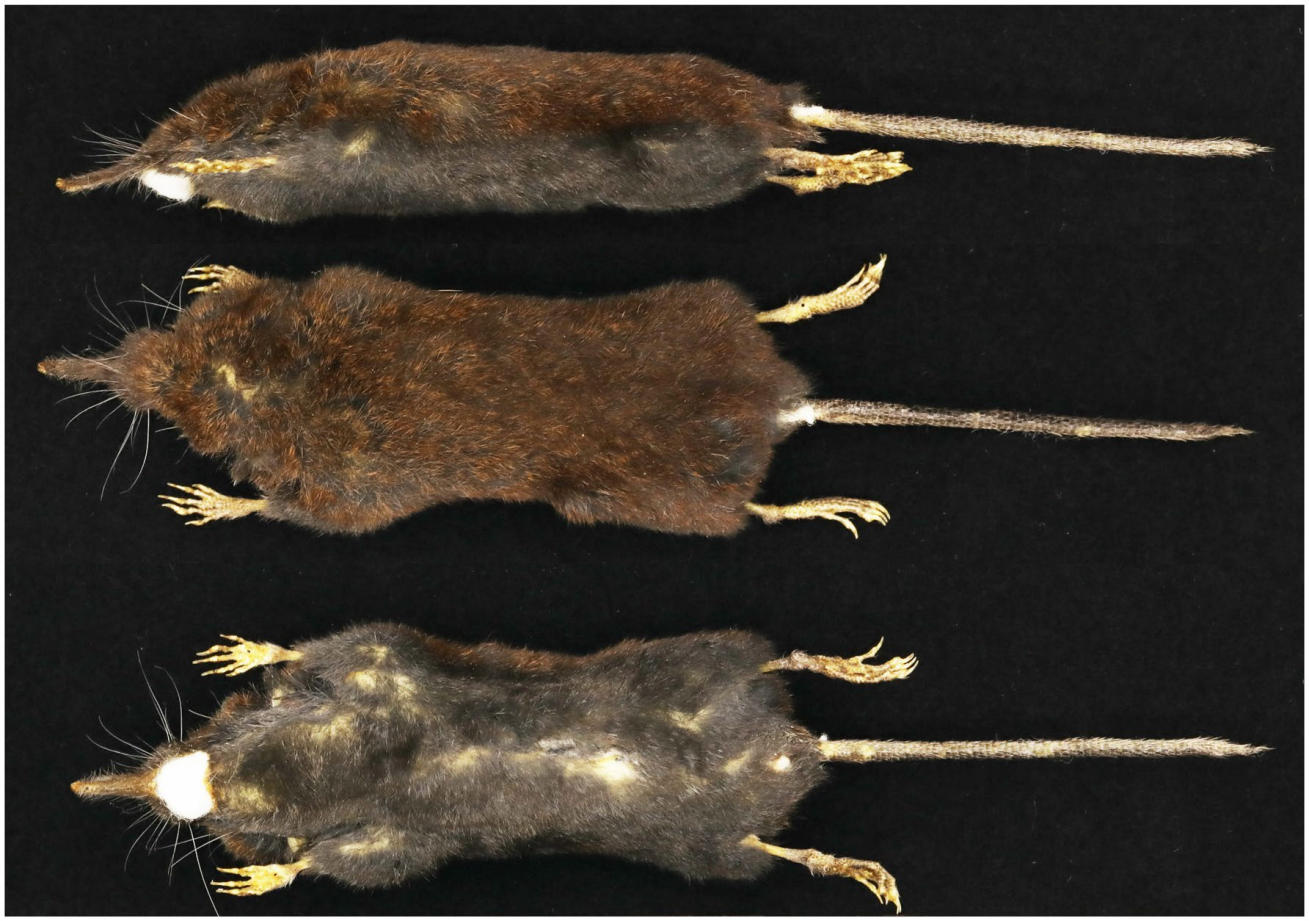
Lateral, dorsal, and ventral views of *U*. *funiushanensis* sp. nov.

**FIGURE 6:**
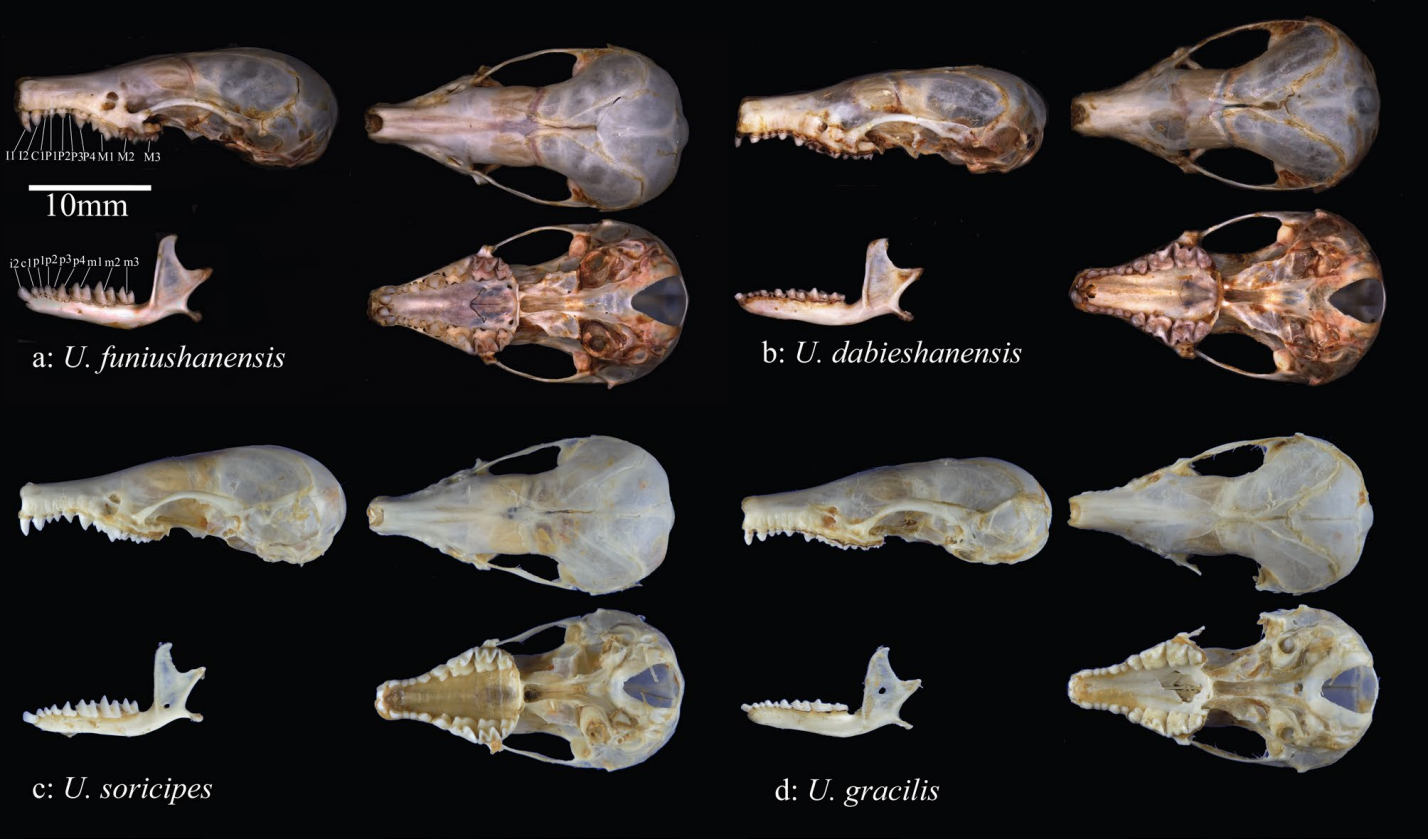
Dorsal, ventral, and lateral views of the skull and lateral views of the mandible for *Uropsilus* species (a *U. funiushanensis* sp. nov; b *U. dabieshanensis*; c *U. soricipes* ; d *U. gracilis*).

Body bicolor, dorsum yellowish–brown, and venter ash black, with a distinct separation between the dorsum and venter (Figure 5). The tail is relatively short, approximately 80% of the head and body length. The tufts at the tail tip are short. The snout is short and robust. The zygomatic arch is slender, exhibiting a distinct rounded curvature from the ventral skull. The lacrimal foramina are similar in size. Dental formula $I\frac{2}{1},C\frac{1}{1}P\frac{4}{4},M\frac{3}{3}=38$ . The buccal view of the upper fourth premolar (P4) and lower fourth premolar (p4) triangular in shape. The first lower premolar (p1) is slightly larger than the third lower premolar (p3). The lower canine (c1) is larger than p1. The upper canine and first premolars (C1 and P1, respectively) are approximately the same size. The first incisor 1 (I1) is larger than I2; both are closely aligned with no noticeable gaps, and I1 is inwardly angled toward I2 (Figure 6).

### 4.3 Description

*U*. *funiushanensis* sp. nov. is a medium‐sized shrew mole (hbl = 73.1 ± 3.4, tl = 59.4 ± 3.2, hl = 14.3 ± 0.6, PL = 20.74 ± 0.44, Tables S6 and S7). The dorsal pelage is yellowish–brown, with the tips of the dorsal fur being brownish–yellow and the bases being gray. The ventral surface is grayish–black. The tail is short and robust. The dorsal part of the tail is black, whereas the ventral side is lighter. The hind foot length (hl = 14.3 ± 0.6 mm) constitutes approximately 19.56% of the combined head and body length.

The shape of the skull is elliptical, featuring a fully intact zygomatic arch. The rostrum is short and robust, with a relatively wide maxilla. The zygomatic arches are slender and slightly bowed outward. The lacrimal foramen and infraorbital foramen are similar in size. The foramen magnum is relatively large. The least breadth between the orbits is small. The median palatal process is short. Both the upper and lower dental rows are shorter.

The dental formula of *U. funiushanensis* sp. nov. is $I\frac{2}{1},C\frac{1}{1}P\frac{4}{4},M\frac{3}{3}=38$. I1 is larger than I2, with no gaps between them. C1 is slightly smaller than P1, and P3 is the smallest; the upper premolars are ordered in size as P4 > P2 > P1 > P3. M1 and M2 are larger than M3; all of them exhibit W‐shaped lateral cusps. Among the mandibular teeth, the size order is i1 > c1 > p1. Among the lower premolars, the size order is p4 > p2 > p1 > p3, with p4 being triangular and featuring a sharp cusp. For the lower molars, the size order is m2 > m1 > m3.

### 4.4 Distribution and Ecological Notes

*U*. *funiushanensis* sp. nov. is currently documented from Shennongjia, Hubei Province, as well as Xiaoqinling, Yaoshan, Tianchishan, Yanzishan, and Longyuwan in Henan Province, central China. The altitude range spans from 889 to 2100 m. This new species inhabited diverse habitats, including broadleaved forest, coniferous forest with a humus layer, coniferous–broadleaved forest, and bamboo forest.

### 4.5 Comparison

The phylogenetic relationships indicate that *U*. *funiushanensis* sp. nov. and *U. soricipes* group ( *U. soricipes* and *U*. sp. 5) were sister lineages (Figure 3a,b). Among the other species in genus *Uropsilus*, the closest geographical neighbors to *U. funiushanensis* sp. nov. were *U. dabieshanensis* and *U*. sp. 5. *U. funiushanensis* sp. nov. is totally distinguished from other species of *Uropsilus* by some distinctive characteristics.

Compared to *U. soricipes* , *U. funiushanensis* sp. nov. can be easily distinguished based on dental formula. The dental formula of *U. funiushanensis* sp. nov. is $I\frac{2}{1},C\frac{1}{1},P\frac{4}{4},M\frac{3}{3}=38$, while the dental formula of *U. soricipes* is $I\frac{2}{1},C\frac{1}{1},P\frac{3}{3},M\frac{3}{3}=34$ (Figure 6). The structure of the third premolar (P3 and p3) of *U*. *funiushanensis* sp. nov. is prominently and easily observed, while it is completely absent in *U. soricipes* . The BL and LBO of *U. funiushanensis* sp. nov. are smaller than *U. soricipes* (Table S6). The ML of *U. funiushanensis* sp. nov. is longer, while the LMTR is smaller than *U. soricipes* . The zygomatic arch of *U. funiushanensis* sp. nov. is distinctly curved, while the *U. soricipes* approaches a straight line.

Compared to *U. dabieshanensis*, the head and body length of *U. funiushanensis* sp. nov. was shorter (hbl = 73.1 ± 3.4 mm vs. hbl = 74.4 ± 3.8 mm). The tail of *U. funiushanensis* sp. nov. displayed dichromatic coloration, while *U. dabieshanensis* is almost uniform. The tail length of *U. funiushanensis* sp. nov. is bigger than *U. dabieshanensis* (tl = 59.4 ± 3.2 mm vs. tl = 54.2 ± 4.1 mm). Additionally, the tail of *U*. *funiushanensis* sp. nov. (tl/bl = 81%) was notably larger than *U. dabieshanensis* (tl/bl = 73%) in percentage (Table S7). Both zygomatic arches are curved; *U. dabieshanensis* is more pronounced than *U. funiushanensis* sp. nov. Both the dental formula of *U. funiushanensis* sp. nov. and *U. dabieshanensis* were $I\frac{2}{1},C\frac{1}{1},P\frac{4}{4},M\frac{3}{3}=38$. However, there are distinguishing features between the two species. For instance, the I1 and I2 are closely connected with no gaps in *U. funiushanensis* sp. nov., whereas *U. dabieshanensi* exhibited a distinguishable gap (Figure 6). *U*. *funiushanensis* sp. nov. was smaller than *U. dabieshanensis* (LBO, MPL, UTRL, LTRL, and M^2^–M^2^).

Compared to *U. gracilis* , the head and body length of *U. funiushanensis* sp. nov. were significantly larger. The tail length of *U. funiushanensis* sp. nov. was notably shorter than that of *U. gracilis* , and the ratio of tl/hbl is comparatively smaller (81 vs. 104, Table S7). The skull of *U. funiushanensis* sp. nov. was smaller than *U. gracilis* (Table S6). The zygomatic arch of *U. gracilis* was thicker than that of *U*. *funiushanensis* sp. nov. On the ventral side the zygomatic arch of *U*. *funiushanensis* sp. nov. appeared more curved, while the lateral shows greater curvature in *U. gracilis*. Both the dental formula of *U*. *funiushanensis* sp. nov. and *U. gracilis* were $I\frac{2}{1},C\frac{1}{1},P\frac{4}{4},M\frac{3}{3}=38$. There was no gap between I1 and I2 in *U*. *funiushanensis* sp. nov., whereas *U. gracilis* exhibits a gap (Figure 6).

The dental formula of *U. funiushanensis* sp. nov. is identical to that of *U. investigator*, *U. huanggangensis*, *U. fansipanensis*, *U. atronates*, and *U. nivatus* ($I\frac{2}{1},C\frac{1}{1},P\frac{4}{4},M\frac{3}{3}=38$). The PL of *U*. *funiushanensis* sp. nov. (PL = 20.74 ± 0.44 mm) is smaller than *U. investigator* (PL = 21.10 ± 0.44 mm) (Hu et al. 2021; Ren et al. 2023). The color difference in the dorsal and ventral hair of *U*. *funiushanensis* sp. nov. was clearly distinguished, while *U. investigator* cannot be distinguished by eyes. The dorsal pelage of *U*. *funiushanensis*. sp. nov. (yellowish–brown) differs from *U. atronate*s (chestnut red) and *U*. *nivatus* (black–gold). And the claybank in back of *U*. *funiushanensis* sp. nov. was more obvious than *U*. *huanggangensis* and *U*. *fansipanensis*.

The dental formula of *U*. *funiushanensis* sp. nov. ($I\frac{2}{1},C\frac{1}{1},P\frac{4}{4},M\frac{3}{3}=38$) was different with *U. andersoni* ($I\frac{2}{2},C\frac{1}{1},P\frac{4}{3},M\frac{3}{3}=38$) and *U*. *aequodonenia* ($I\frac{2}{2},C\frac{1}{1},P\frac{3}{3},M\frac{3}{3}=36$), which can be easily distinguished among these species (Table S1).

## 5 Discussion

Shrew moles have long been known to inhabit the mountainous regions of southwestern China and neighboring Myanmar (Kryštufek and Motokawa 2018; Wan 2015; Wan, He, and Jiang 2013; Wan et al. 2018). In recent studies, several new species of *Uropsilus* have been reported from different regions, which greatly expanding their distribution range. For example, Hu et al. (2021) expanded the known distribution of the genus *Uropsilus* by reporting the presence of *U*. *dabieshanensis* in the Mt. Dabieshan, Anhui Province, central China. Bui et al. (2023) expanded the known distribution of the genus *Uropsilus* by reporting the presence of *U*. *fansipanensis* in Vietnam. Moreover, Ren et al. (2023) contributed to the report of *U*. *huanggangensis* from the Mt. Huanggang, Jiangxi Province, east China. However, no records of *Uropsilus* have been reported from Henan Province, central China. In this study, we formally recognize *U*. *funiushanensis* as a new species from Shennongjia, Hubei Province, and western mountains of Henan Province based on integrative taxonomic studies. Importantly, these specimens collected from western mountains in Henan Province could represent the northernmost record of the genus *Uropsilus*, thereby bridging the distributional gap between the Mts. Qinling and Dabieshan areas.

The phylogenetic studies of *Uropsilus* have utilized mitochondrial or combined with nuclear gene datasets, lacking comprehensive analyses of nuclear gene phylogenetic relationships (Hu et al. 2021; Bui et al. 2023; Ren et al. 2023). In this study, different datasets reveal distinct and unstable phylogenetic relationships, particularly in the nuDNA dataset (RAG1, RAG2, and PLCB4) (Wan, He, and Jiang 2013). Based on the PLCB4, most species of the genus *Uropsilus* are clustered together, with a few species distinguishable (Figure S1c). Based on the RAG2, *U. funiushanensis* is intermingled with the *U. soricipes* group (Figure S1b). Based on the RAG1, distinct lineages for the various species of *Uropsilus* are identified, and *U. funiushanensis* forms a sister clade with *U. dabieshanensis* + *U*. sp. 3 + *U*. sp. 4. However, phylogenetic relationships inferred from a single nuclear gene sequence are insufficient to provide a clear and comprehensive understanding of the evolutionary history of *Uropsilus* (Figure S1). These results are due to incomplete lineage sorting or gene flow. Based on the BI (CYT B and nuDNA) and species delimitation (ASAP and BPP) results, *U*. *funiushanensis* is classified as a distinct species. Furthermore, most species within the genus *Uropsilus* are clearly delineated, whereas the taxonomic classification of *U*. sp. 1 and *U*. sp. 7 requires further investigation. The divergence time of *U*. *funiushanensis* is estimated in early Pleistocene (2.16 Ma, 95% CI: 2.00–2.65). The global cooling and drying events during the Pleistocene epoch facilitated the rapid speciation of genus *Uropsilus* (Hu et al. 2021; Ren et al. 2023; Wan 2015; Wan, He, and Jiang 2013; Wan et al. 2018). Based on molecular analyses, we conclude specimens of *Uropsilus* collected from Henan and Hubei Provinces represent the same new species, *U*. *funiushanensis*. Unfortunately, the specimen has sustained significant damage from Shennongjia in Hubei Province, which made it difficult to ascertain the morphological characteristics (Wan 2015). Further sampling and analysis are required to determine whether the morphological characteristics of specimens from Hubei and Henan Provinces.

Due to the intricate topography of the southwestern mountains, some species of Eulipotyphla have formed small and isolated genetic populations (Chen et al. 2017; He and Jiang 2014; Wan, He, and Jiang 2013; Wan et al. 2018). The species of *Uropsilus* in the Mt. Qinling are consistent with this genetic pattern. The dental formula of *U*. *funiushanensis* is consistent with *U. gracilis* groups (Table S1), while the phylogenetic relationships are related to *U. soricipes* groups (Figure 3). In future studies, more attention should be paid to investigating the evolutionary mechanisms underlying the dental formula within *Uropsilus*.

Recently, several new species of Eulipotyphla have been described in central and eastern China, including *Crocidua dongyangjiangensis* (Liu et al. 2020), *Uropsilus dabieshanensis* (Hu et al. 2021), *Chodsigoa dabieshanensis* (Chen et al. 2022), *Parablarinella latimaxillata* (Chen et al. 2023), *Uropsilus huanggangensis* (Ren et al. 2023), and *Mesechinus orientalis* (Shi et al. 2023). Based on the findings of these new species, it can be inferred that there is additional cryptic biodiversity in central and eastern China. Notably, Henan Province is located in central and eastern China, encompassing the Mts. Taihangshan, Funiushan, and Tongbaishan–Dabieshan, which provide potential habitats for small mammals (Lu and Wang 2012). We believe that the species diversity of small mammals in Henan Province has likely been underestimated, which highlights the necessity for increased attention and further investigation.

## Author Contributions

**Haijun Jiang:** data curation (lead), formal analysis (lead), investigation (lead), software (lead), writing – original draft (lead), writing – review and editing (equal). **Yaohua Yang:** data curation (equal), investigation (equal). **Yanyan Zhou:** formal analysis (equal), software (equal). **Jundong Tian:** project administration (equal), supervision (equal). **Jiqi Lu:** funding acquisition (lead), project administration (lead), resources (lead), supervision (lead), writing – review and editing (lead).

## Conflicts of Interest

The authors declare no conflicts of interest.

## Supporting information


Figure S1.

Figure S2.

Figure S3.

Tables S1–S7.

## Data Availability

New DNA sequences in this study were deposited in GenBank (Accession numbers : CYT B PP430752 – PP430769; PLCB4 PP430770 – PP430788; RAG1 PP430789 – PP430806; RAG2 PP430807 – PP430824 PP430752–P430824) .

## References

[ece370928-bib-0001] Allen, G. M. 1923. “New Chinese Insectivores.” American Museum Novitates 100: 1–11. http://hdl.handle.net/2246/4530.

[ece370928-bib-0002] Allen, G. M. 1938. The Mammals of China and Mongolia. New York: American Museum of Natural History.

[ece370928-bib-0003] Bui, H. T. , S. Okabe , L. T. H. Le , N. T. Nguyen , and M. Motokawa . 2023. “A New Shrew Mole Species of the Genus *Uropsilus* (Eulipotyphla: Talpidae) From Northwestern Vietnam.” Zootaxa 5339, no. 1: 59–78. 10.11646/zootaxa.5339.1.3. 38221066

[ece370928-bib-0004] Chen, Z. Z. , K. He , C. Huang , et al. 2017. “Integrative Systematic Analyses of the Genus *Chodsigoa* (Mammalia: Eulipotyphla: Soricidae), With Descriptions of New Species.” Zoological Journal of the Linnean Society 180, no. 3: 694–713. 10.1093/zoolinnean/zlw017.

[ece370928-bib-0005] Chen, Z. Z. , J. X. Hu , K. He , et al. 2023. “Molecular and Morphological Evidence Support a New Species of Asiatic Short‐Tailed Shrew (Eulipotyphla: Soricidae).” Journal of Mammalogy 104, no. 6: 1455–1467. 10.1093/jmammal/gyad087.

[ece370928-bib-0006] Chen, Z. Z. , T. Hu , X. Pei , et al. 2022. “A New Species of Asiatic Shrew of the Genus *Chodsigoa* (Soricidae, Eulipotyphla, Mammalia) From the Dabie Mountains, Anhui Province, Eastern China.” ZooKeys 1083: 129–146. 10.3897/zookeys.1083.78233. 35115875 PMC8807571

[ece370928-bib-0007] Darriba, D. , G. L. Taboada , R. Doallo , and D. Posada . 2012. “jModelTest 2: More Models, New Heuristics and Parallel Computing.” Nature Methods 9, no. 8: 772. 10.1038/nmeth.2109. PMC4594756 22847109

[ece370928-bib-0008] Douady, C. J. , and E. J. Douzery . 2003. “Molecular Estimation of Eulipotyphlan Divergence Times and the Evolution of “Insectivora”.” Molecular Biology and Evolution 28, no. 2: 285–296. 10.1016/s1055-7903(03)00119-2. 12878465

[ece370928-bib-0009] Ellerman, J. , and T. Morrison‐Scott . 1951. “Checklist of Palaearctic and Indian Mammals 1758 to 1946. London, British Museum (Natural History).” Science 115: 431–432. 10.1126/science.115.2990.431.

[ece370928-bib-0010] He, K. , and X. L. Jiang . 2014. “Sky Islands of Southwest China. I: An Overview of Phylogeographic Patterns.” Chinese Science Bulletin 59, no. 7: 585–597. 10.1007/s11434-013-0089-1.

[ece370928-bib-0011] He, K. , A. Shinohara , K. M. Helgen , M. S. Springer , X. L. Jiang , and K. L. Campbell . 2017. “Talpid Mole Phylogeny Unites Shrew Moles and Illuminates Overlooked Cryptic Species Diversity.” Molecular Biology and Evolution 34, no. 1: 78–87. 10.1093/molbev/msw221. 27795230

[ece370928-bib-0012] Hoffmann, R. S. 1984. “A Review of the Shrew‐Moles (Genus *Uropsilus*) of China and Burma.” Journal of the Mammalogical Society of Japan 10: 69–80. 10.11238/JMAMMSOCJAPAN1952.10.69.

[ece370928-bib-0013] Hu, T. L. , Z. Xu , H. Zhang , et al. 2021. “Description of a New Species of the Genus *Uropsilus* (Eulipotyphla: Talpidae: Uropsilinae) From the Dabie Mountains, Anhui, Eastern China.” Zoological Research 42, no. 3: 294–299. 10.24272/j.issn.2095-8137.2020.266. 33929104 PMC8175959

[ece370928-bib-0014] Hutterer, R. 2005. “Order Soricomorpha.” In Mammal Species of the World: A Taxonomic and Geographic Reference. Vol. 2, edited by E. D. Wilson and M. D. Reeder , 3rd ed. Baltimore, MD: Johns Hopkins University Press.

[ece370928-bib-0015] Jiang, H. J. , C. K. Fu , K. Y. Tang , et al. 2023. “Molecular Phylogenetics and Diversity of the Himalayan Shrew (*Soriculus nigrescens* Gray, 1842) (Eulipotyphla, Soricidae) in Southwest China.” Zootaxa 5263, no. 1: 61–78. 10.11646/zootaxa.5263.1.3. 37044999

[ece370928-bib-0016] Kimura, M. 1980. “A Simple Method for Estimating Evolutionary Rates of Base Substitutions Through Comparative Studies of Nucleotide Sequences.” Journal of Molecular Evolution 16, no. 2: 111–120. 10.1007/BF01731581. 7463489

[ece370928-bib-0017] Kryštufek, B. , and M. Motokawa . 2018. “Family Talpidae (Moles, Desmans, Star‐Nosed Moles and Shrew Moles).” Handbook of the Mammals of the World 8: 552–619.

[ece370928-bib-0018] Liu, Y. , S. D. Chen , B. Q. Liu , R. Liao , Y. X. Liu , and S. Y. Liu . 2020. “A New Species of the Genus *Crocidura* (Eulipotyphla: Soricidae) From Zhejiang Pvovince, Eastern China.” Acta Theriologica Sinica 40, no. 1: 1–12. 10.16829/j.slxb.150340.

[ece370928-bib-0019] Liu, Y. , S. Y. Liu , Z. Y. Sun , P. Guo , Z. X. Fan , and W. M. Robert . 2013. “A New Species of *Uropsilus* (Talpidae:Uropsilinae) From Sichuan, China.” Acta Theriological Sinica 33, no. 2: 113–122.

[ece370928-bib-0020] Lu, J. Q. , and Z. L. Wang . 2012. Glires Fauna and Ecology in Henan Province, China. Zhengzhou, China: Zhengzhou University Press.

[ece370928-bib-0021] Milne‐Edwards, A. 1871. “Descriptions of New Species, in Footnotes, in David Journal d'un Voyage en Mongolia et en Chine Fait en 1866–68.” Nouvelles Archives du Muséum D'Histoire Naturelle 7: 75–100.

[ece370928-bib-0022] Motokawa, M. 2004. “Phylogenetic Relationships Within the Family Talpidae (Mammalia: Insectivora).” Journal of Zoology 263: 147–157. 10.1017/S0952836904004972.

[ece370928-bib-0023] Puillandre, N. , S. Brouillet , and G. Achaz . 2021. “ASAP: Assemble Species by Automatic Partitioning.” Molecular Ecology Resources 21, no. 2: 609–620. 10.1111/1755-0998.13281. 33058550

[ece370928-bib-0024] R Core Team . 2022. R: A Language and Environment for Statistical Computing. Vienna, Austria: R Foundation for Statistical Computing.

[ece370928-bib-0025] R Studio Team . 2024. “RStudio: Integrated Development Environment for R (Version 2024.04.2).” https://posit.co/download/rstudio/.

[ece370928-bib-0026] Rambaut, A. , A. J. Drummond , D. Xie , G. Baele , and M. A. Suchard . 2018. “Posterior Summarization in Bayesian Phylogenetics Using Tracer 1.7.” Systematic Biology 67, no. 5: 901–904. 10.1093/sysbio/syy032. 29718447 PMC6101584

[ece370928-bib-0027] Ren, X. , Y. Xu , Y. Li , et al. 2023. “A New Species of Shrew Moles, Genus *Uropsilus* Milne‐Edwards, 1871 (Mammalia, Eulipotyphla, Talpidae), from the Wuyi Mountains, Jiangxi Province, Eastern China.” Zookeys 1186: 25–46. 10.3897/zookeys.1186.111592. 38107661 PMC10724864

[ece370928-bib-0028] Sanchez‐Villagra, M. R. , I. Horovitz , and M. Motokawa . 2006. “A Comprehensive Morphological Analysis of Talpid Moles (Mammalia) Phylogenetic Relationships.” Cladistics 22, no. 1: 59–88. 10.1111/j.1096-0031.2006.00087.x. 34892894

[ece370928-bib-0029] Shi, Z. F. , H. F. Yao , K. He , et al. 2023. “A New Species of Forest Hedgehog (Mesechinus, Erinaceidae, Eulipotyphla, Mammalia) From Eastern China.” ZooKeys 1185: 143–161. 10.3897/zookeys.1185.111615. 38074901 PMC10698749

[ece370928-bib-0030] Shinohara, A. , K. L. Campbell , and H. Suzuki . 2003. “Molecular Phylogenetic Relationships of Moles, Shrew Moles, and Desmans From the New and Old Worlds.” Molecular Biology and Evolution 27, no. 2: 247–258. 10.1016/s1055-7903(02)00416-5. 12695089

[ece370928-bib-0031] Smith, A. , and Y. Xie . 2008. A Guide to the Mammals of China. Princeton, NJ: Princeton University Press.

[ece370928-bib-0032] Suchard, M. A. , P. Lemey , G. Baele , D. L. Ayres , A. J. Drummond , and A. Rambaut . 2018. “Bayesian Phylogenetic and Phylodynamic Data Integration Using BEAST 1.10.” Virus Evolution 4, no. 1: vey016. 10.1093/ve/vey016. 29942656 PMC6007674

[ece370928-bib-0033] Tamura, K. , G. Stecher , and S. Kumar . 2021. “MEGA11 Molecular Evolutionary Genetics Analysis Version 11.” Molecular Biology and Evolution 38, no. 7: 3022–3027. 10.1093/molbev/msab120. 33892491 PMC8233496

[ece370928-bib-0034] Thomas, O. 1912. “The Duke of Bedford's Zoological Exploration of Eastern Asia.—XV. On Mammals From the Provinces of Szechwan and Yunnah, Western China.” Proceedings of the Zoological Society of London 82, no. 1: 127–141. 10.1111/j.1469-7998.1912.tb07008.x.

[ece370928-bib-0035] Thomas, O. 1922. “On Mammals From the Yunnan Highlands Collected by Mr. George Forrest and Presented to the British Museum by Col. Stephenson R. Clarke D. S. O.” Annals and Magazine of Natural History 10, no. 58: 391–406. 10.1080/00222932208632789.

[ece370928-bib-0036] Wan, T. 2015. Phylogeny, Phylogeography and Integrative Taxonomy of Asiatic Shrew Moles (Uropsilinae). Beijing, China: University of Chinese Academy of Sciences.

[ece370928-bib-0037] Wan, T. , K. He , and X. L. Jiang . 2013. “Multilocus Phylogeny and Cryptic Diversity in Asian Shrew‐Like Moles (Uropsilus, Talpidae): Implications for Taxonomy and Conservation.” BMC Evolutionary Biology 13: 232. 10.1186/1471-2148-13-232. 24161152 PMC3819745

[ece370928-bib-0038] Wan, T. , K. He , W. Jin , et al. 2018. “Climate Niche Conservatism and Complex Topography Illuminate the Cryptic Diversification of Asian Shrew‐Like Moles.” Journal of Biogeography 45, no. 10: 2400–2414. 10.1111/jbi.13401.

[ece370928-bib-0039] Wang, Y. , and G. Yang . 1989. Editor‐in‐Chief of Yunnan Disease Control Office and Yunnan Health and Anti‐Epidemic Station: Yunnan Medical Animal Directory. Kunming, China: Yunnan Science and Technology Press.

[ece370928-bib-0040] Wei, F. W. , Q. S. Yang , Y. Wu , et al. 2021. “Catalogue of Mammals in China.” Acta Theriologica Sinica 41, no. 5: 487–501. 10.16829/j.slxb.150595.

[ece370928-bib-0041] Yang, Q. , L. Xia , Z. Feng , Y. Ma , G. Quan , and Y. Wu . 2007. “A Guide to the Measurement of Mammal Skull V: Insectivora and Chiroptera.” Chinese Journal of Zoology 42, no. 2: 56–62. 10.13859/j.cjz.2007.02.013.

[ece370928-bib-0042] Yang, Q. , L. Xia , Y. Ma , Z. J. Feng , and G. Q. Quan . 2005. “A Guide to the Measurement of Mammal Skull I: Basic Measurement.” Chinese Journal of Zoology 40, no. 3: 50–56. 10.13859/j.cjz.2005.03.011.

[ece370928-bib-0043] Yang, Z. , and B. Rannala . 2014. “Unguided Species Delimitation Using DNA Sequence Data From Multiple Loci.” Molecular Biology and Evolution 31, no. 12: 3125–3135. 10.1093/molbev/msu279. 25274273 PMC4245825

