# Supplementary figures and images for: Molecular Phylogeny of Uropsilus (Talpidae, Eulipotyphla, Mammalia) With a New Species Described From Henan Province, China
Source: Ecol Evol. 2025 Feb 12;15(2):e70928. doi: 10.1002/ece3.70928 (PMC11814535; doi:10.1002/ece3.70928)

a: RAG1

0.002

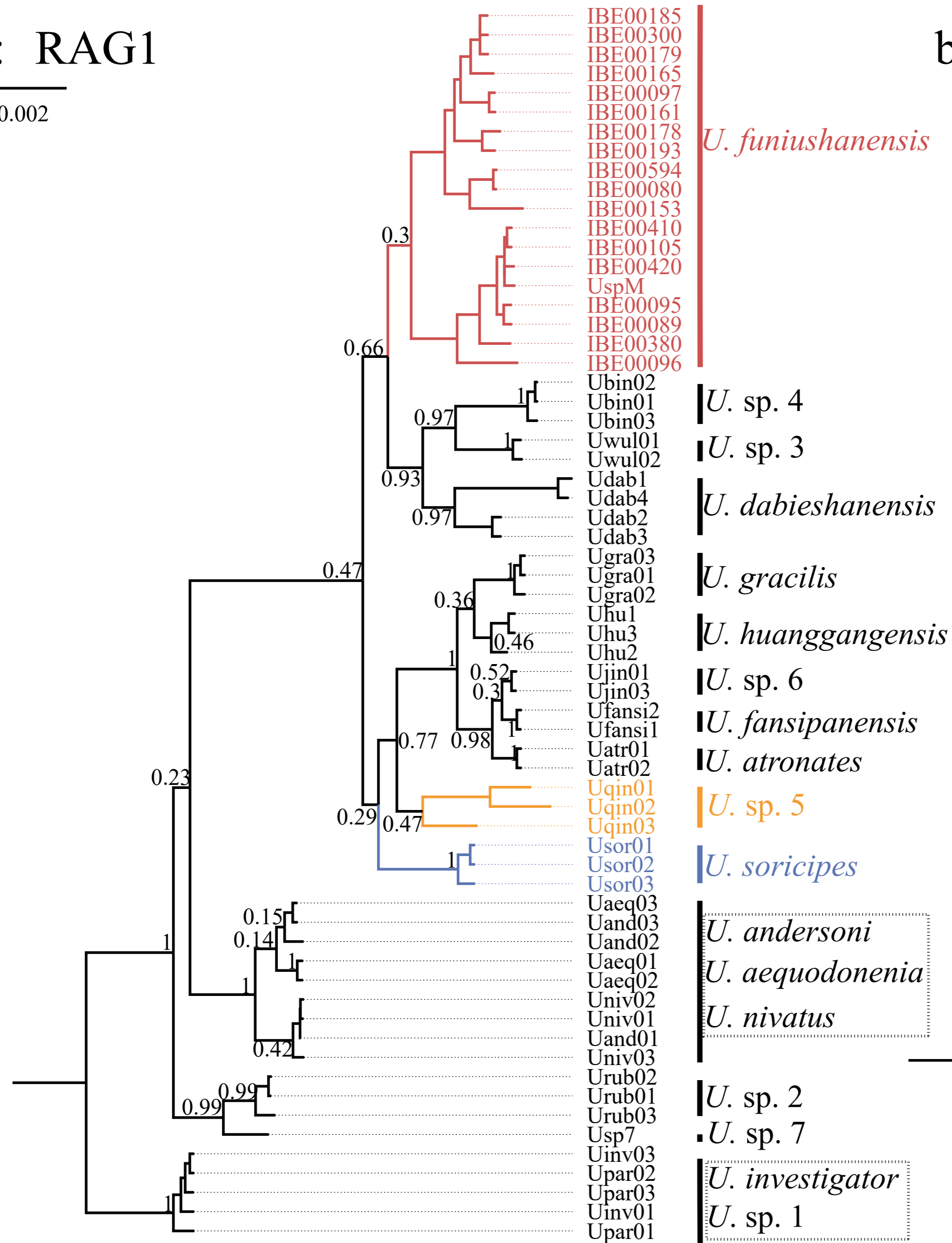

b: RAG2

0.001

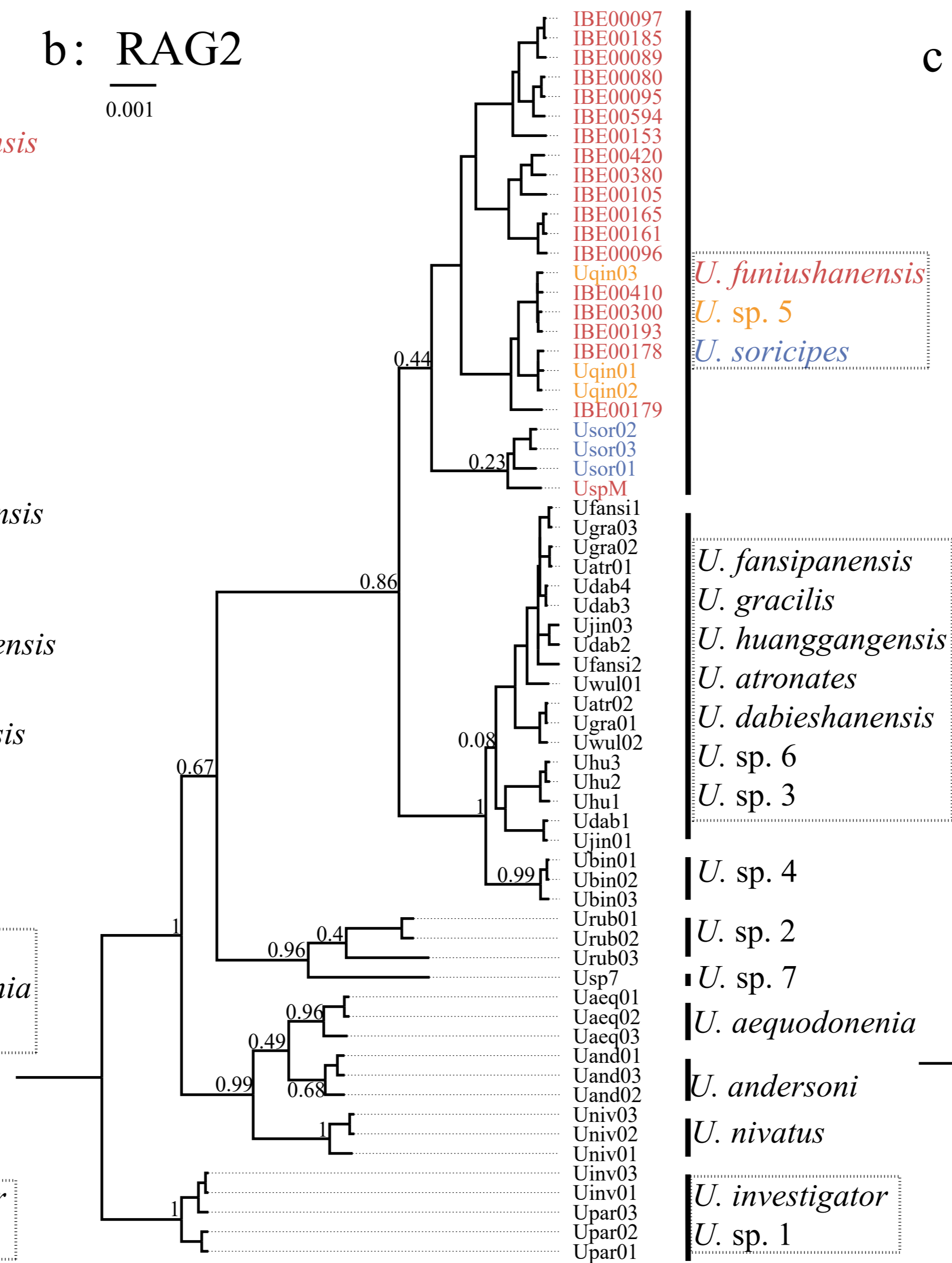

c: PLCB4

0.002

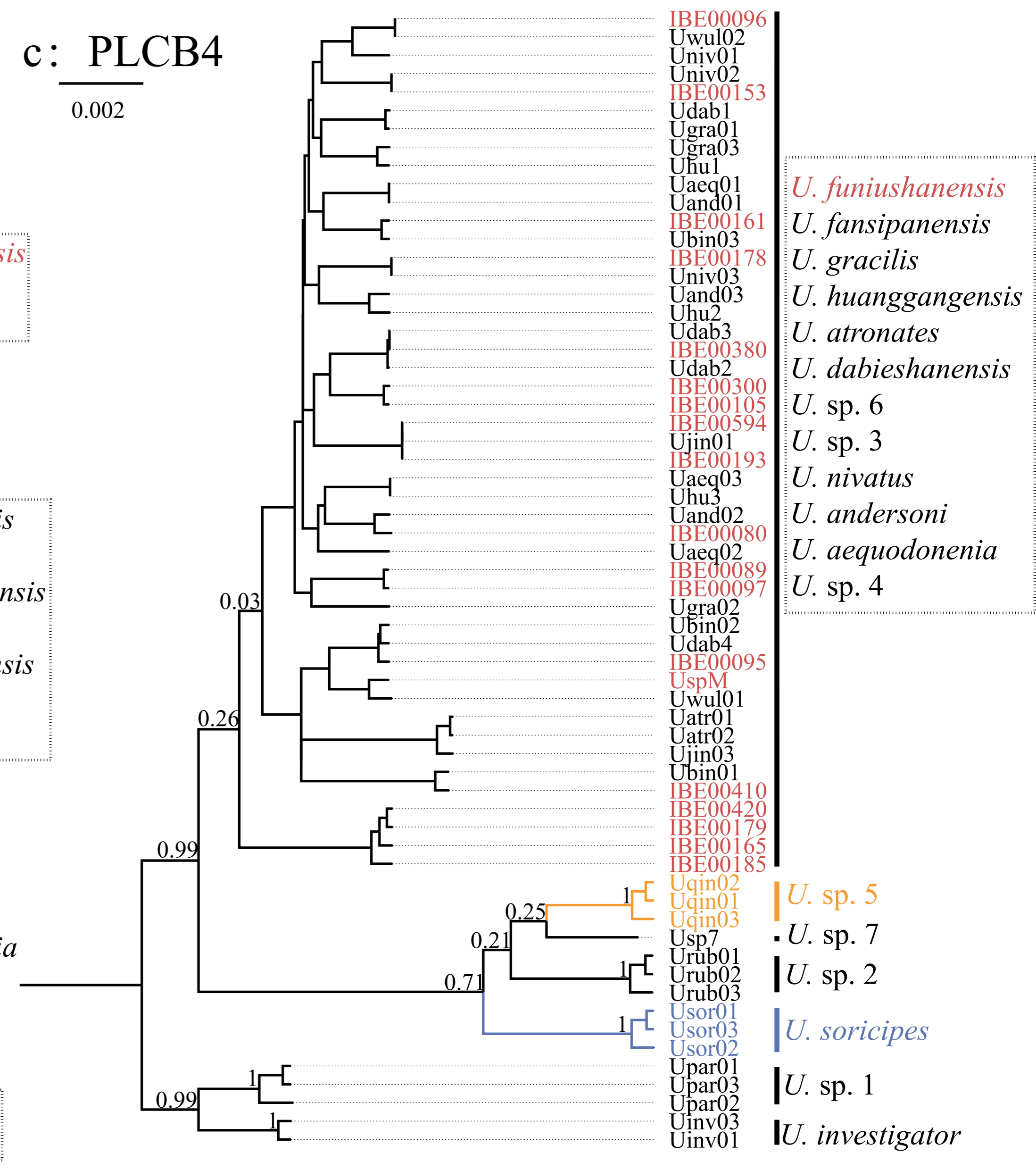

Supplement: Supplementary file 1 — Figure S1. [file ECE3-15-e70928-s002.pdf]
