# Supplementary material for: Molecular Phylogeny of Uropsilus (Talpidae, Eulipotyphla, Mammalia) With a New Species Described From Henan Province, China
Source: Ecol Evol. 2025 Feb 12;15(2):e70928. doi: 10.1002/ece3.70928 (PMC11814535; doi:10.1002/ece3.70928)
Supplement: Supplementary file 4 — Tables S1–S7. [file ECE3-15-e70928-s004.docx]

ATTACHMENTS
Table S1 The dental formulas of the genus *Uropsilus.*

|  | dental formula | | | |
| --- | --- | --- | --- | --- |
|  | $I\frac{2}{1}, C\frac{1}{1}, P\frac{3}{3}, M\frac{3}{3}$ = 34 | $I\frac{2}{2}, C\frac{1}{1}, P\frac{3}{3}, M\frac{3}{3}$ = 36 | $I\frac{2}{2}, C\frac{1}{1}, P\frac{4}{3}, M\frac{3}{3}$ = 38 | $I\frac{2}{1}, C\frac{1}{1}, P\frac{4}{4}, M\frac{3}{3}$ = 38 |
| species | *U. soricipes*  *U.* sp *.*5 | *U. aequodonenia* | *U. andersoni* | *U. gracilis*  *U. nivatus*  *U. atronates*  *U. investigator*  *U. dabieshanensis*  *U. huanggangensis*  *U. fansipanensis*  *U*. sp. 1  *U*. sp. 2  *U*. sp. 3  *U*. sp. 4  *U*. sp. 6  *U. funiushanensis* sp. nov. |
| incisors (I), canines (C), premolars (P), molars (M) | | | | |

Table S2 Sampling localities of *U. funiushanensis* sp. nov. in Henan Province.

| Sample site | voucher number | location | longitude | latitude | Altitude (m) |
| --- | --- | --- | --- | --- | --- |
| 1 | IBE00080 | Tianchishan of Songxian, Henan province | 111.84 | 34.265 | 1107.55 |
|  | IBE00089 | Tianchishan of Songxian, Henan province | 111.8217 | 34.26282 | 1243.05 |
|  | IBE00095 | Tianchishan of Songxian, Henan province | 111.8217 | 34.26282 | 1243.05 |
|  | IBE00096 | Tianchishan of Songxian, Henan province | 111.8217 | 34.26282 | 1243.05 |
|  | IBE00097 | Tianchishan of Songxian, Henan province | 111.8217 | 34.26282 | 1243.05 |
|  | IBE00105 | Tianchishan of Songxian, Henan province | 111.8217 | 34.26282 | 1243.05 |
| 2 | IBE00153 | Longyuwan of Luanchuan, Henan province | 111.7562 | 33.70335 | 1079.49 |
|  | IBE00161 | Longyuwan of Luanchuan, Henan province | 111.7941 | 33.67037 | 1675.02 |
|  | IBE00165 | Longyuwan of Luanchuan, Henan province | 111.7893 | 33.66407 | 1969.43 |
|  | IBE00178 | Longyuwan of Luanchuan, Henan province | 111.7919 | 33.67459 | 1622.62 |
|  | IBE00179 | Longyuwan of Luanchuan, Henan province | 111.7919 | 33.67459 | 1622.62 |
|  | IBE00185 | Longyuwan of Luanchuan, Henan province | 111.7919 | 33.67459 | 1622.62 |
|  | IBE00193 | Longyuwan of Luanchuan, Henan province | 111.7898 | 33.66486 | 1927.12 |
| 3 | IBE00300 | Yanzishan of Lingbao, Henan province | 111.0956 | 34.50765 | 889.18 |
| 4 | IBE00380 | Xiaoqinling of Lingbao, Henan province | 110.5712 | 34.41439 | 1408.07 |
|  | IBE00410 | Xiaoqinling of Lingbao, Henan province | 110.5787 | 34.40873 | 1612.2 |
|  | IBE00420 | Xiaoqinling of Lingbao, Henan province | 110.5787 | 34.40873 | 1612.2 |
| 5 | IBE00594 | Yaoshan of Lushan, Henan province | 112.2462 | 33.71968 | 2100 |

Table S3 PCR and sequencing primers used in this study (refer to Wan, 2015).

| Locus | Primer name | Primer sequences (5’→3’) |
| --- | --- | --- |
| CYT B | L14724_hk3 | GGACTTATGACATGAAAAATCATCGTTG |
|  | H15915_hk3 | GATTCCCCATTTCTGGTTTACAAGAC |
| PLCB4 | PLCB4-F | GTGAAATTGGAAGCCGAGAT |
|  | PLCB4-R | CACCAAGCTCATTTACTTGTGA |
| RAG1 | F1705 | GCTTTGATGGACATGGAAGAAGACAT |
|  | R2951 | GAGCCATCCCTCTCAATAATTTCAGG |
| RAG2 | RAG2-F220 | GATTCCTGCTAYCTYCCTCCTCT |
|  | RAG2-R995 | CCCATGTTGCTTCCAAACCATA |

Table S4 Sequence information of GenBank accession numbers for species used in this study.

| Species | Specimen voucher | Identification code | GenBank Accession Number | | | |
| --- | --- | --- | --- | --- | --- | --- |
|  |  |  | CYT B | RAG1 | RAG2 | PLCB4 |
| *U. investigator* | KIZ-028527 | Uinv01 | KF778154 | KF778274 | KF778367 | KF778215 |
|  | KIZ-201211136 | Uinv03 | MH209680 | KF778271 | KF778363 | MH210124 |
| *U. gracilis* | KIZ-028544 | Ugra01 | KF778208 | KF778312 | KF778368 | KF778260 |
|  | KIZ-028545 | Ugra02 | KF778209 | KF778311 | KF778369 | KF778259 |
|  | KIZ-028546 | Ugra03 | KF778206 | KF778313 | KF778371 | KF778252 |
| *U. soricipes* | KIZ-028553 | Usor01 | KF778191 | KF778317 | KF778350 | KF778225 |
|  | KIZ-028554 | Usor02 | KF778192 | KF778318 | KF778351 | KF778226 |
|  | KIZ-028555 | Usor03 | KF778193 | KF778319 | KF778374 | KF778224 |
| *U. aequodonenia* | SAF-07940 | Uaeq01 | MH209698 | MH210253 | MH210378 | MH210144 |
|  | SAF-04010 | Uaeq02 | MH209699 | MH210254 | MH210379 | MH210145 |
|  | KIZ-0906042 | Uaeq03 | MH209766 | MH210315 | MH210424 | MH210193 |
| *U. andersoni* | KIZ-Z201405738 | Uand01 | MH209675 | MH210232 | MH210354 | MH210118 |
|  | KIZ-Z201405739 | Uand02 | MH209676 | MH210233 | MH210355 | MH210119 |
|  | SAF-06948 | Uand03 | MH209738 | MH210288 | MH210413 | MH210173 |
| *U. nivatus* | KIZ-022473 | Univ01 | KF778168 | KF778291 | KF778356 | KF778247 |
|  | KIZ-022469 | Univ02 | KF778166 | KF778292 | KF778359 | KF778248 |
|  | KIZ-022974 | Univ03 | KF778170 | KF778293 | KF778360 | KF778249 |
| *U. atronates* | KIZ-028531 | Uatr01 | KF778180 | KF778303 | KF778332 | KF778238 |
|  | KIZ-028532 | Uatr02 | KF778184 | KF778304 | KF778333 | KF778237 |
| *U.* sp. 6 | KIZ-019509 | Ujin01 | KF778187 | KF778301 | KF778324 | KF778233 |
|  | KIZ-019517 | Ujin03 | KF778188 | KF778307 | KF778323 | KF778236 |
| *U.* sp. 1 | KIZ-020527 | Upar01 | KF778159 | KF778274 | KF778367 | KF778219 |
|  | KIZ-020539 | Upar02 | KF778160 | KF778275 | KF778331 | KF778216 |
|  | KIZ-028526 | Upar03 | KF778156 | KF778271 | KF778363 | KF778220 |
| *U.* sp. 4 | KIZ-BC1110001 | Ubin01 | MH209643 | MH210202 | MH210324 | MH210087 |
|  | KIZ-BC1110012 | Ubin02 | MH209644 | MH210203 | MH210325 | MH210088 |
|  | KIZ-BC1110129 | Ubin03 | MH209645 | MH210204 | MH210326 | MH210089 |
| *U.* sp. 2 | KIZ028549 | Urub01 | KF778161 | KF778280 | KF778376 | KF778212 |
|  | KIZ028550 | Urub02 | KF778162 | KF778277 | KF778375 | KF778213 |
|  | KIZ028557 | Urub03 | KF778164 | KF778278 | KF778328 | KF778211 |
| *U.* sp. 5 | KIZ016096 | Uqin01 | KF778195 | KF778266 | KF7783339 | KF778261 |
|  | KIZ016101 | Uqin02 | KF778199 | KF778276 | KF7783340 | KF778262 |
|  | KIZ016109 | Uqin03 | KF778196 | KF778314 | KF7783341 | KF778265 |
| *U.* sp. 3 | SAF-06040 | Uwul01 | MH209692 | MH210247 | MH210371 | MH210136 |
|  | SAF-06042 | Uwul02 | MH209693 | MH210248 | MH210372 | MH210137 |
| *Usp*M | KIZ-C204112 | UspM | MH209691 | MH210246 | MH210370 | MH210135 |
| *U.* sp. 7 | SAF-12098 | Usp7 | MH209720 | MH210270 | MH210395 | MH210160 |
| *U. dabieshanensis* | AE1612YLP017 | Udab1 | MT199125 | MT211605 | MT211608 | MW321508 |
|  | AE1807FZL007 | Udab2 | MT199126 | MT211606 | MT211609 | MW321509 |
|  | AE1907FZL001 | Udab3 | MT199127 | MT211607 | MT211610 | MW321510 |
|  | AE2005YLP001 | Udab4 | MT710697 | MT710698 | MT710699 | MW321511 |
| *U. huanggangensis* | Ahnu202206013 | Uhu1 | OQ730193 | OQ730198 | OQ730203 | OR161365 |
|  | Ahnu202206014 | Uhu2 | OQ730194 | OQ730199 | OQ730204 | OR161366 |
|  | Ahnu202206053 | Uhu3 | OQ730195 | OQ730200 | OQ730205 | OR161367 |
| *U. fansipanensis* | IEBR-M-8101 | Ufansi1 | OQ509373 | OQ509371 | OQ512024 | NA |
|  | IEBR-M-8102 | Ufansi2 | OQ509374 | OQ509372 | OQ512025 | NA |

Table S5 Best molecular evolution models for phylogenetic reconstructions according to AIC.

| Gene | model |
| --- | --- |
| CYT B | HKY+I+G |
| PLCB4 | HKY |
| RAG1 | HKY+I |
| RAG2 | HKY+I |

Table S6 Skull measurements (mm) used in morphometric analyses of *Uropsilus*.

|  | *U. dabieshanensis* (*n* = 4) | *U. gracilis*  (*n* = 5) | *U. soricipes* (*n* = 7) | *U.* sp. 5 (*n* =3) | *U*. *funiushanensis*  sp. nov.  (n = 12) |
| --- | --- | --- | --- | --- | --- |
| PL | 19.75–21.24 | 22.00–22.80 | 20.25–21.60 | 20.91–21.75 | 20.04–21.4 |
|  | 20.63±0.64 | 22.21±0.34 | 20.92±0.48 | 21.23±0.45 | 20.74±0.44 |
| BL | 16.68–17.53 | 17.25–18.40 | 16.40–18.20 | 16.69–17.54 | 16.06–17.8 |
|  | 17.09±0.39 | 17.71±0.42 | 17.21±0.62 | 17.09±0.43 | 16.95±0.37 |
| LBO | 5.05–5.30 | 4.81–5.50 | 5.20–5.60 | 4.77–5.03 | 4.08–5.00 |
|  | 5.19±0.11 | 5.03±0.27 | 5.37±0.37 | 4.84±0.14 | **4.48±0.25** |
| HB | 6.26–6.70 | 7.00–7.35 | 6.20–7.20 | 7.07–7.82 | 6.46–7.48 |
|  | 6.49±0.21 | 7.20±0.15 | 6.80±0.34 | 7.49±0.38 | 6.86±0.33 |
| MPL | 9.66–10.71 | 10.00–10.70 | 9.35–10.20 | 9.75–10.24 | 9.00–10.04 |
|  | 10.04±0.26 | 10.33±0.27 | 9.75±0.35 | 10.00±0.25 | **9.61±0.31** |
| ZB | 10.48–10.71 | 10.90–11.35 | 10.30–11.40 | 10.57–11.06 | 10.10–11.26 |
|  | 10.61±0.10 | 11.16±0.19 | 10.75±0.37 | 10.89±0.27 | 10.63±0.33 |
| UTRL | 9.23–9.73 | 9.75–10.20 | 8.80±9.60 | 9.38–9.59 | 8.74–9.62 |
|  | 9.47±0.24 | 9.97±0.18 | 9.23±0.28 | 9.46±0.11 | **9.15±0.27** |
| M^2^-M^2^ | 6.76–6.99 | 6.55–6.95 | 6.20–6.80 | 6.50–7.02 | 6.42–7.10 |
|  | 6.91±0.10 | 6.77±0.15 | 6.56±0.22 | 6.78±0.26 | **6.67±0.20** |
| ML | 14.15–14.71 | 14.50–15.45 | 13.10–14.05 | 14.36–14.00 | 13.54–15.02 |
|  | 14.38±0.26 | 14.77±0.39 | 13.57±0.40 | 14.22±0.19 | 14.12±0.39 |
| LTRL | 8.32–8.72 | 8.90–9.50 | 7.95–8.60 | 8.53–8.74 | 7.80–8.54 |
|  | 8.52±0.20 | 9.15±0.23 | 8.32±0.23 | 8.62±0.11 | **8.24±0.23** |
| PL: Profile length; BL: Basal length; LBO: least breadth between the orbits; ZB: Zygomatic breadth; HB: Height of braincase; MPL: Median palatal length; UTRL: Upper tooth row length; M^2^–M^2^: Greatest width measured at anterior labial margins of second mandibular; ML: Mandibular length; LTRL: Lower of tooth row length. | | | | | |

Table S7 Comparison of external morphological data for *U. funiushanensis* sp. nov., *U. dabieshanensis* and *U. soricipes.*

|  | *U. funiushanensis*  sp.nov (n = 18) | *U. dabieshanensis*  (n = 13) | *U.* sp. 5  (n = 21) | *U. soricipes*  (n = 7) | *U. gracilis*  (*n* = 5) |
| --- | --- | --- | --- | --- | --- |
| hbl (mm) | **66 – 78****, 73.1** **± 3.4** | 65 - 83, 74.4 ± 3.8 | 66 - 80, 71.2 ± 3.6 | 60 - 75, 69.38 ± 4.53 | 70 - 75, 72.2 ± 1.79 |
| tl (mm) | **53 – 63, 59.4 ± 3.2** | 45 - 59, 54.2 ± 4.1 | 60 - 70, 64.1 ± 2.6 | 58 - 68, 63.63 ± 3.42 | 70 - 83,75.4 ± 4.72 |
| hl (mm) | **13 – 16, 14.3 ± 0.6** | 12 - 15,13.9 ± 0.6 | 13 - 14,13.4 ± 0.5 | 13 - 15,14.13 ± 0.83 | 14 - 15, 14.8 ± 0. 45 |
| tl/hbl (%) | **81** | 73 | 90 | 92 | 104 |
| hbl: head and body length; tl: tail length; hl: hindfoot length | | | | | |
