# Supplementary material for: Molecular Phylogeny of Uropsilus (Talpidae, Eulipotyphla, Mammalia) With a New Species Described From Henan Province, China
Source: Ecol Evol. 2025 Feb 12;15(2):e70928. doi: 10.1002/ece3.70928 (PMC11814535; doi:10.1002/ece3.70928)
Supplement: Supplementary file 2 — Figure S2. [file ECE3-15-e70928-s003.pdf]

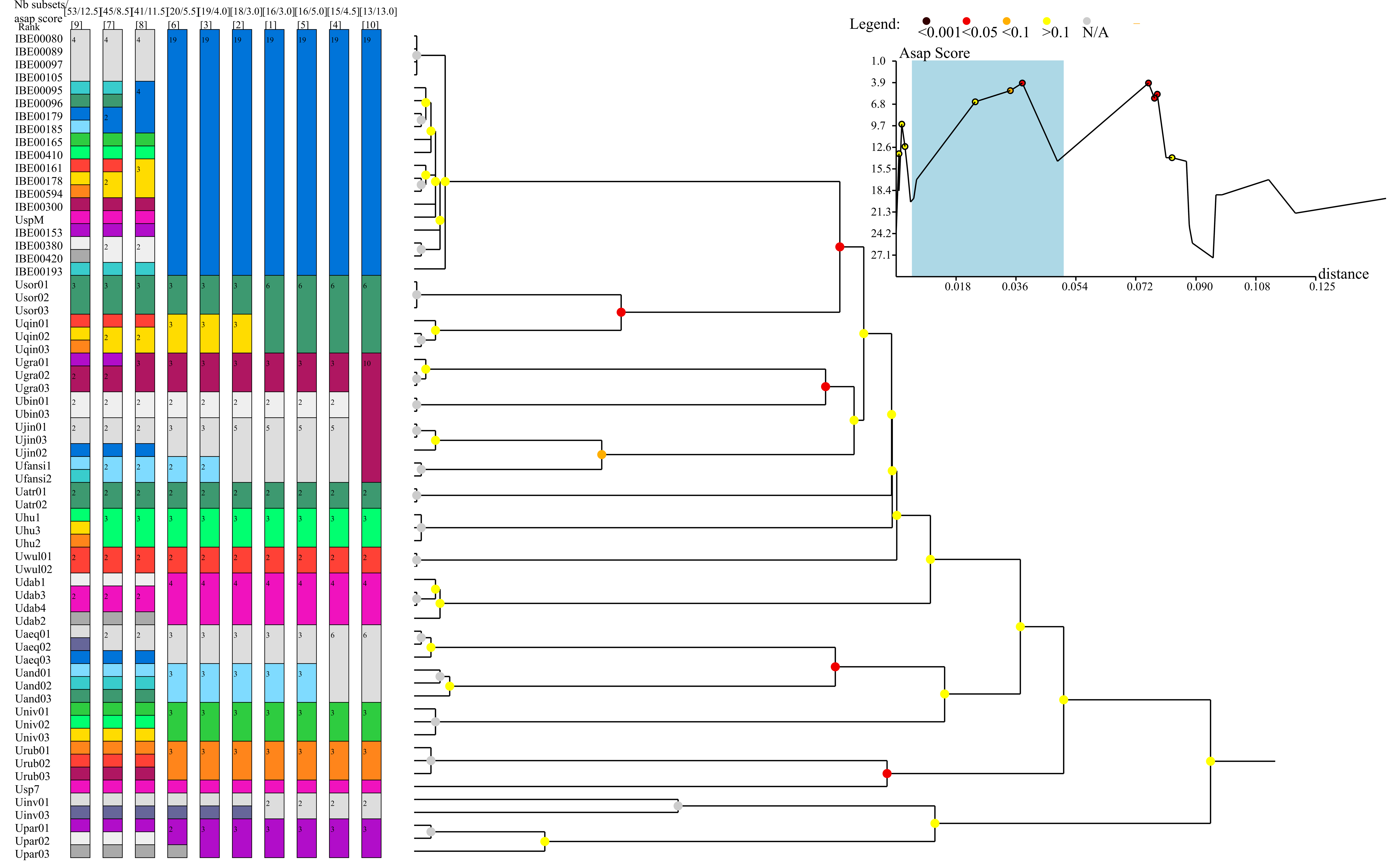
Nb subsets/
asap score
Rank
[53/12.5] [45/8.5] [41/11.5] [20/5.5] [19/4.0] [18/3.0] [16/3.0] [16/5.0] [15/4.5] [13/13.0]
[9] [7] [8] [6] [3] [2] [1] [5] [4] [10]
IBE00080
IBE00089
IBE00097
IBE00105
IBE00095
IBE00096
IBE00179
IBE00185
IBE00165
IBE00410
IBE00161
IBE00178
IBE00594
IBE00300
UspM
IBE00153
IBE00380
IBE00420
IBE00193
Usor01
Usor02
Usor03
Uqin01
Uqin02
Uqin03
Ugra01
Ugra02
Ugra03
Ubin01
Ubin03
Ujin01
Ujin03
Ujin02
Ufansi1
Ufansi2
Uatr01
Uatr02
Uhu1
Uhu3
Uhu2
Uwul01
Uwul02
Udab1
Udab3
Udab4
Udab2
Uaeq01
Uaeq02
Uaeq03
Uand01
Uand02
Uand03
Univ01
Univ02
Univ03
Urub01
Urub02
Urub03
Usp7
Uinv01
Uinv03
Upar01
Upar02
Upar03
Legend:
<0.001 <0.05 <0.1 >0.1 N/A
Asap Score
1.0
3.9
6.8
9.7
12.6
15.5
18.4
21.3
24.2
27.1
0.018
0.036
0.054
0.072
0.090
0.108
0.125
distance
