# Supplementary material for: Molecular Phylogeny of Uropsilus (Talpidae, Eulipotyphla, Mammalia) With a New Species Described From Henan Province, China
Source: Ecol Evol. 2025 Feb 12;15(2):e70928. doi: 10.1002/ece3.70928 (PMC11814535; doi:10.1002/ece3.70928)
Supplement: Supplementary file 3 — Figure S3. [file ECE3-15-e70928-s001.pdf]

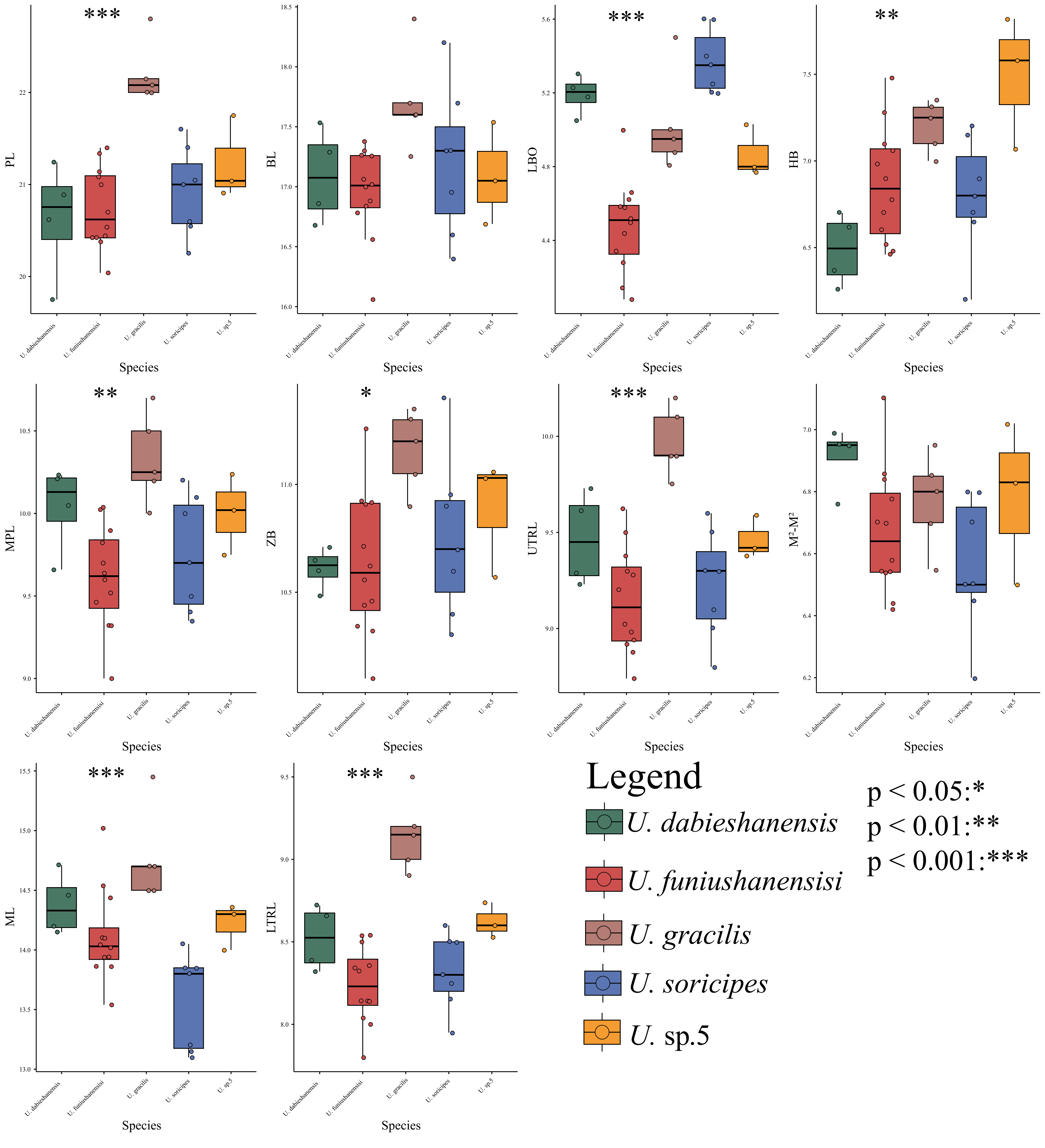

PL
***
BL
LBO
***
HB
**
MPL
**
ZB
*
UTRL
***
M²-M²
ML
***
LTRL
***
Species
U. dabieshanensis
U. funiushanensisi
U. gracilis
U. soricipes
U. sp.5
Legend
U. dabieshanensis
U. funiushanensisi
U. gracilis
U. soricipes
U. sp.5
p < 0.05:*
p < 0.01:**
p < 0.001:***
